# Overcoming Therapy Resistance in Ovarian Cancer: From Molecular Mechanisms to Emerging Therapeutic Strategies

**DOI:** 10.3390/cancers18162623

**Published:** 2026-08-14

**Authors:** Zofia Pietrasik, Mikołaj Kapała, Joanna Pietrasik, Monika Stefaniak, Sebastian Szubert, Krzysztof Książek, Justyna Mikuła-Pietrasik

**Affiliations:** 1 Department of Pathophysiology of Aging and Civilization Diseases, Poznan University of Medical Sciences, Swiecickiego 4 Str., 60-781 Poznan, Poland; zofia.pietrasik02@gmail.com (Z.P.); apietrasik2002@gmail.com (J.P.); 2 Medical University of Łódź, Al. T. Kościuszki 4 Str., 90-419 Lodz, Poland; mikolajkapala1@gmail.com; 3 Institute of Health Sciences, Faculty of Medical Sciences, Prince Mieszko I Poznań Medical University of Applied Sciences, Bułgarska 55 Str., 60-320 Poznan, Poland; monika.stefaniak@interia.pl (M.S.); k.ksiazek@pam.poznan.pl (K.K.); 4 Department of Gynecological Oncology, Institute of Oncology, University of Medical Sciences, 82/84 Szamarzewskiego Str., 60-569 Poznan, Poland; sszubert@ump.edu.pl

**Keywords:** CAR-T, chemoresistance, epigenetic therapies, HIPEC, immunotherapy, metabolic therapies, ovarian cancer, PIPAC, targeted therapies, tumor microenvironment

## Abstract

**Background/Objectives**: Epithelial ovarian cancer (EOC) remains a gynecologic malignancy with a poor prognosis, with a 5-year survival of approximately 29% in advanced-stage disease. Despite cytoreductive surgery and platinum- and taxane-based chemotherapy, most patients relapse within 2 years. Major therapeutic barriers include chemoresistance, molecular heterogeneity, and an immunosuppressive peritoneal microenvironment. This review summarizes emerging therapeutic strategies for EOC, their mechanisms of action, and their potential to overcome treatment resistance. **Methods**: PubMed/MEDLINE was searched for preclinical studies, phase I–III clinical trials, systematic reviews, and meta-analyses addressing novel ovarian cancer therapies and resistance mechanisms. **Results:** The review covers molecularly targeted therapies, immunotherapies, metabolic and epigenetic approaches, cellular and gene therapies, targeted drug-delivery systems, and locoregional and physical modalities. Strategies include PARP inhibitors, antiangiogenic agents, antibody–drug conjugates, pathway inhibitors, immune checkpoint inhibitors, cancer vaccines, adoptive cell therapies, metabolic and epigenetic modulators, CAR-T, CAR-NK, CRISPR/Cas9, HIPEC, PIPAC, ablation, photodynamic therapy, and sonodynamic therapy. **Conclusions**: The clinical maturity of these approaches varies substantially. PARP inhibitors, antiangiogenic agents, selected antibody–drug conjugates, MAPK-directed therapy in LGSOC, and HIPEC in selected settings have the strongest clinical support. Most immune combinations, metabolic and epigenetic therapies, adoptive cell therapies, gene-editing approaches, and novel delivery or physical modalities remain early clinical or predominantly preclinical. Progress will depend on biomarker-guided patient selection, reassessment of evolving resistance mechanisms, and rational treatment sequencing and combinations.

## Simple Summary

Ovarian cancer remains a major therapeutic challenge because many patients eventually experience disease recurrence and develop resistance to available treatments. Although recent therapeutic advances have improved outcomes in selected patient populations, there remains an urgent need for effective strategies to prevent or overcome treatment resistance. In this review, we summarize recent developments in ovarian cancer therapy, with particular emphasis on approaches targeting tumor-intrinsic biology, antitumor immunity, cancer metabolism, epigenetic regulation, and emerging cellular, genetic, and physical treatment modalities. We examine how these strategies may restore therapeutic sensitivity, delay disease progression, and improve clinical outcomes. Finally, we highlight personalized treatment selection, biomarker development, and rational combination therapies as key priorities for future research and clinical translation in ovarian cancer.

## 1. Introduction

Epithelial ovarian cancer (EOC) is one of the most common and, at the same time, most lethal gynecologic malignancies. The lifetime risk of developing the disease is 1 in 75, while the lifetime risk of death is 1 in 100 [1]. In the vast majority of cases, the disease is diagnosed at an advanced clinical stage, at which the 5-year survival rate is less than 30% [1,2]. Most malignant ovarian tumors are, in fact, of extraovarian origin. Data from risk-reducing salpingo-oophorectomy programs involving BRCA1/2 mutation carriers have demonstrated a high prevalence of fallopian tube carcinoma or its precursor, serous tubal intraepithelial carcinoma (STIC), leading to a revision of the histogenesis of high-grade serous carcinoma [1,3,4]. The vast majority of both benign and malignant ovarian tumors arise from epithelial, stromal, or germ cells, with epithelial tumors accounting for as many as 90% of cases in developed countries [1,5].

Ovarian carcinomas comprise a heterogeneous group of malignancies encompassing histologic subtypes with distinct pathogenetic mechanisms, molecular profiles, gene expression patterns, and prognoses. Five major subtypes account for approximately 90% of all cases: high-grade serous ovarian carcinoma (HGSOC; ~70%), endometrioid ovarian carcinoma (EC; ~10%), clear cell ovarian carcinoma (CCC; ~10%), low-grade serous ovarian carcinoma (LGSOC; ~5%), and mucinous ovarian carcinoma (MC; ~3–4%) [1,5,6].

The epidemiology of EOC shows marked geographic variation, with higher incidence rates observed in North America and Central and Eastern Europe, and lower rates in Asia and Africa [2,7]. These patterns have changed dynamically in recent decades: incidence has declined in Northern Europe and North America while increasing in Eastern Europe and Asia [1,2,7]. The observation that migration from low-incidence areas to high-incidence regions is associated with an increased risk of developing the disease indicates an important role for environmental and lifestyle factors in its etiology [7].

EOC biology is characterized by a marked predilection for dissemination within the peritoneal cavity. Tumor progression may follow two distinct pathways: the gradual accumulation of mutations, progressing from a slow-growing borderline tumor to a well-differentiated carcinoma (type I), or the development of an aggressive, genetically unstable high-grade serous carcinoma that gives rise to peritoneal metastases at an early stage (type II) [3,4]. Ovarian cancer cells undergo epithelial–mesenchymal transition (EMT), characterized by changes in the expression of cadherins, vimentin, and integrins, along with increased activity of proteolytic pathways. Tumor cell spheroids transported by peritoneal fluid evade anoikis and preferentially adhere to the peritoneum or greater omentum, where they undergo mesenchymal–epithelial transition (MET), thereby reacquiring an epithelial phenotype that enables colonization of new sites [3].

The aim of this review is to present the current state of knowledge on contemporary therapeutic strategies for EOC, with particular emphasis on their potential to prevent disease recurrence and overcome mechanisms of treatment resistance. The terms emerging/novel therapeutic strategies in ovarian cancer refer to approaches that extend beyond standard management based on cytoreductive surgery and platinum- and taxane-based chemotherapy. It encompasses both methods already used in routine clinical practice for selected indications and interventions currently under preclinical or clinical investigation. Their common objectives are to improve treatment efficacy, reduce the risk of disease recurrence, and support the development of more precise and personalized therapeutic approaches.

In the following sections, we discuss the principal categories of contemporary therapeutic strategies for EOC, with particular attention to their mechanisms of action, the available clinical evidence, and their relevance to treatment resistance.

## 2. Materials and Methods

This structured narrative review was based primarily on searches of PubMed/MEDLINE covering publications from 2000 to 2026. The final search was conducted on 15 July 2026. The search combined the terms “ovarian cancer” or “epithelial ovarian cancer” with terms related to treatment resistance and individual therapeutic modalities, including “targeted therapy,” “immunotherapy,” “PARP inhibitors,” “antiangiogenic therapy,” “metabolic therapy,” “epigenetic therapy,” “nanomedicine,” “photodynamic therapy,” “sonodynamic therapy,” “irreversible electroporation,” “CAR-T,” “HIPEC,” “PIPAC,” “chemoresistance,” and “tumor microenvironment.” Reference lists of relevant reviews and pivotal clinical trials were also screened manually.

Only publications in English were considered. When evaluating clinical efficacy and applicability, priority was given to randomized controlled trials, meta-analyses, peer-reviewed clinical studies, regulatory documents, and current clinical guidelines. Phase I–II trials and observational studies were included to assess early clinical activity and safety, whereas preclinical studies were used primarily to describe biological mechanisms, therapeutic rationale, and investigational strategies.

Clinical guidelines and regulatory documents were used to establish the current clinical status, indications, and maturity of individual therapeutic approaches rather than as substitutes for primary efficacy data. Case reports were included only when they illustrated an emerging therapeutic concept for which higher-level evidence was unavailable and were explicitly interpreted as exploratory evidence. Preprints and conference abstracts were considered only when no full peer-reviewed publication was available and when they provided information directly relevant to an emerging strategy or ongoing clinical study. Such sources were clearly identified in the text, and conclusions based on them were interpreted cautiously.

Studies involving multiple tumor types were included only when ovarian cancer-specific results were reported or when they addressed a directly relevant biological mechanism or shared barrier to clinical translation that had not yet been adequately evaluated in EOC. Evidence derived from other malignancies was used primarily to discuss general limitations of therapeutic classes, including delivery efficiency, metabolic plasticity, immune effects, tumor heterogeneity, pharmacologic constraints, or manufacturing challenges. Such evidence was not interpreted as demonstrating therapeutic efficacy in EOC, and its indirect nature was explicitly acknowledged where relevant.

Titles and abstracts were independently screened by two authors, followed by full-text assessment of potentially relevant publications. Disagreements were resolved through discussion or, when necessary, consultation with a third author. Because this was a narrative review, no formal risk-of-bias assessment or quantitative synthesis of evidence was performed.

When interpreting the available evidence, therapeutic strategies were categorized according to their stage of clinical development and evidentiary maturity. Approaches supported by regulatory approval, guideline recommendations, or randomized phase III trials were considered clinically established or clinically validated in selected populations. Strategies supported mainly by phase I–II trials or nonrandomized clinical studies were classified as early clinical. Approaches evaluated predominantly in vitro, in animal models, in conference reports, preprints, or isolated case reports were considered preclinical, exploratory, or hypothesis-generating, as appropriate.

The review is limited by its primary reliance on a single bibliographic database, its restriction to English-language publications, its non-systematic study selection, the heterogeneity of the included evidence, and the possibility of selection and publication bias. Additional limitations arise from the inclusion of selected conference abstracts, preprints, case reports, clinical guidelines, and studies involving other tumor types. Although these sources were used selectively to capture emerging concepts and shared translational barriers, they provide lower- or indirect-level evidence and should not be considered equivalent to ovarian cancer-specific, peer-reviewed clinical data.

## 3. Results

### 3.1. Molecular Basis of EOC

#### Molecular Heterogeneity of HGSOC Compared with Other Histologic Subtypes

The molecular heterogeneity of EOC represents a fundamental diagnostic and therapeutic challenge (Table 1). This problem exists at two levels: interpatient heterogeneity, reflecting differences between patients, and intratumoral heterogeneity, resulting from the coexistence of distinct cellular clones within a single tumor. This diversity explains why tumors with the same histologic diagnosis and FIGO stage may exhibit markedly different treatment responses and clinical outcomes [8,9].

HGSOC is characterized by the near-universal presence of TP53 mutations (96%), a high frequency of somatic copy-number alterations, and whole-genome duplications [1,3]. Low-frequency but statistically significant somatic mutations in NF1, BRCA1, BRCA2, RB1, and CDK12 occur in 5–8% of tumors [3,10,11]. A key feature of HGSOC is homologous recombination deficiency (HRD), which is present in approximately 50% of cases. HRD results from germline or somatic BRCA1/2 mutations, collectively found in approximately 25% of HGSOCs; mutations in other DNA repair pathway genes, including CHEK2, RAD51, BRIP1, and PALB2; or epigenetic inactivation of BRCA1 through promoter methylation [1,3,11,12,13]. The most common amplifications involve CCNE1, MYC, and MECOM, whereas recurrent deletions affect RB1, NF1, and PTEN [10,11].

At the transcriptomic level, HGSOC can be stratified into four molecular subtypes: mesenchymal (C1.MES), immunoreactive (C2.IMM), proliferative (C5.PRO), and differentiated (C4.DIF). These subtypes differ in prognosis, treatment response, and immune profile [8,11,12,14]. The immunoreactive subtype is characterized by a higher frequency of BRCA1/2 mutations and greater immune cell infiltration, whereas the proliferative subtype shows the highest prevalence of CCNE1 amplification and low immune cell infiltration [8,14]. A recent genomic analysis of 640 HGSOC tumors using whole-genome sequencing identified five subtypes based on chromosomal instability (CIN) signatures: two HRD subtypes associated with BRCA1 or BRCA2 alterations and favorable treatment responses, and three homologous recombination-proficient (HRP) subtypes with distinct patterns of structural alterations and differential responses to chemotherapy [15].

Risk factors for EOC vary by histologic subtype and include age, inherited mutations in *BRCA1*, *BRCA2*, *BRIP1*, *RAD51C*, *RAD51D*, *MLH1*, *MSH2*, *MSH6*, and PMS2, reproductive and hormonal factors such as early menarche, late menopause, nulliparity, and obesity, as well as cigarette smoking, physical inactivity, and talc exposure [1,7].

### 3.2. Mechanisms Underlying Therapy Resistance and Heterogeneity of Tumor Cell Responses

#### 3.2.1. Clinical and Biological Definitions of Treatment Resistance

Treatment resistance comprises several clinically and biologically distinct phenomena that should not be used interchangeably. Intrinsic or primary resistance is present before treatment or becomes evident as failure to achieve a meaningful response to initial therapy. It may reflect pre-existing resistant clones or constitutive genetic, epigenetic, phenotypic, and microenvironmental features that reduce treatment sensitivity. Acquired resistance develops after an initial response or period of disease control and emerges under therapeutic selection pressure through expansion of resistant subpopulations or adaptive changes induced during treatment [16].

This distinction also applies to cytotoxic agents other than platinum compounds, including taxanes. Taxane resistance may be intrinsic or may develop after an initial period of sensitivity. However, unlike platinum resistance, taxane resistance in EOC is not routinely classified by a standardized treatment-free interval and is generally described in terms of lack of response, progression during therapy, or loss of previously observed sensitivity. Experimental studies in ovarian cancer confirm that both intrinsic and acquired resistance to taxanes may occur, although the underlying mechanisms and their clinical definitions remain less standardized than for platinum compounds [17].

In EOC, platinum-refractory and clinically platinum-resistant disease represent related but distinct clinical categories. Platinum-refractory disease generally refers to progression during platinum-based chemotherapy or failure to achieve disease control during platinum treatment. Clinically, platinum-resistant recurrence has historically been defined as progression or recurrence within 6 months after completion of the most recent platinum-containing regimen. Recurrence occurring more than 6 months after platinum therapy has conventionally been classified as platinum-sensitive. However, platinum responsiveness exists along a biological and clinical continuum rather than as a strict binary characteristic [18,19].

Persistence, recurrence, and dormancy are also related but non-equivalent concepts. Persistent disease refers to viable malignant disease that is not eradicated by initial treatment and remains detectable or survives as microscopic residual disease. Recurrence denotes the reappearance or renewed progression of disease after a complete clinical response or a period without detectable disease. Dormancy is a biological state in which residual malignant cells or microscopic tumor deposits remain viable but non-proliferative, or in which proliferation is balanced by cell death. Dormant cells may be relatively insensitive to therapies directed against actively dividing cells and may later re-enter the cell cycle, contributing to delayed recurrence. In HGSOC, dormant residual cells and cellular aggregates within the peritoneal cavity may display growth arrest, treatment tolerance, and the capacity for subsequent reattachment and renewed proliferation [20].

The platinum-free interval, defined as the time from the last dose of platinum chemotherapy to documented disease progression, has traditionally been used to estimate the probability of response to a platinum rechallenge. However, the conventional 6-month threshold is an operational clinical classification and does not fully represent the biological continuum of platinum sensitivity. Contemporary recommendations favor recording treatment-free intervals separately from the last platinum therapy, non-platinum therapy, and biological or targeted therapy [19].

This distinction is increasingly important in patients receiving maintenance treatment. PARP inhibitors or bevacizumab may prolong the time from the last platinum dose while continuing to exert therapeutic selection pressure. Consequently, the interval since the last platinum administration may not represent a treatment-free biological period. Treatment decisions should therefore integrate the duration and depth of the previous response, progression during maintenance therapy, complete treatment history, residual toxicity, patient condition, and relevant molecular characteristics rather than rely exclusively on the conventional platinum-free interval [19,21].

#### 3.2.2. Integrated Mechanisms of Resistance to Platinum and Taxane Therapy

Resistance to platinum- and taxane-based chemotherapy in EOC is multifactorial and often reflects the coexistence of altered drug transport, enhanced damage tolerance, impaired cell death signaling, clonal selection, cellular plasticity, and microenvironmental support. These mechanisms frequently interact and may evolve during successive lines of treatment [22,23].

Platinum resistance may result from reduced intracellular drug accumulation caused by impaired uptake or increased efflux, as well as from enhanced detoxification by glutathione and other thiol-containing molecules. Once platinum-induced DNA lesions are formed, their cytotoxic effects may be reduced by increased nucleotide excision repair, increased activity of the Fanconi anemia pathway, restoration or enhancement of homologous recombination, and translesion DNA synthesis. Stabilization and protection of stalled replication forks may provide an additional mechanism of resistance, particularly in BRCA-deficient tumors, by preventing degradation of newly synthesized DNA without necessarily restoring homologous recombination. Resistance is further promoted by impaired activation of apoptosis and other cell-death pathways, allowing malignant cells to tolerate otherwise lethal DNA damage [22,23,24].

Taxane resistance involves reduced intracellular drug exposure and alterations in the microtubule system. Increased ABCB1/P-glycoprotein-mediated efflux can lower intracellular paclitaxel concentrations and may produce cross-resistance to other ABCB1 substrates [25]. Changes in β-tubulin expression or isotype composition, tubulin mutations, altered microtubule polymerization and dynamics, and changes in spindle-assembly-checkpoint signaling may reduce sustained mitotic arrest and permit escape from taxane-induced cell death [26].

These mechanisms do not operate independently. Repeated chemotherapy promotes clonal selection, while epithelial–mesenchymal plasticity, cancer stem-cell properties, autophagy, senescence-associated signaling, and reversible drug-tolerant states may enable temporary survival followed by renewed proliferation. The tumor microenvironment further contributes through hypoxia, extracellular matrix remodeling, soluble cytokines and growth factors, stromal and immune cell interactions, ascites, and metabolic support. These factors may protect malignant cells from both platinum-induced DNA damage and taxane-induced mitotic stress [22,23].

Accordingly, platinum and taxane resistance should be regarded as dynamic and heterogeneous phenotypes rather than consequences of a single molecular alteration. The relative contribution of individual mechanisms may differ among histologic subtypes, metastatic sites, treatment stages, and tumor-cell populations. This complexity limits the predictive value of single biomarkers and supports longitudinal reassessment of evolving resistance, as well as therapeutic strategies targeting complementary mechanisms rather than isolated pathways [22,23].

To connect these resistance phenotypes with the therapeutic categories discussed in the following sections, Table 2 maps the principal mechanisms and therapeutic vulnerabilities to the corresponding treatment strategies, histologic and biomarker-defined contexts, clinical settings, evidence maturity, and major limitations. This framework also illustrates that most emerging interventions do not address a single isolated mechanism of resistance, but rather target interconnected tumor-intrinsic, microenvironmental, immune, metabolic, or locoregional vulnerabilities.

#### 3.2.3. Tumor Microenvironment and the Role of the Extracellular Matrix

The peritoneal microenvironment plays a key role in ovarian cancer progression. It constitutes a complex ecosystem comprising peritoneal mesothelial cells (PMCs), cancer-associated fibroblasts (CAFs), tumor-associated macrophages (TAMs), immune cells, the extracellular matrix (ECM), and soluble signaling factors [114,115,116].

The peritoneal mesothelium is not a passive barrier, but rather a dynamic structure with immunologic, regenerative, and secretory functions. PMCs secrete cytokines, growth factors, and ECM proteins, thereby creating a specialized niche that promotes the adhesion and metastatic dissemination of EOC cells [114,116,117]. Factors such as GRO-1, IL-6, IL-8, and lysophosphatidic acid (LPA) stimulate tumor cell proliferation, migration, and invasion, whereas hyaluronic acid (HA) binds to the CD44 receptor and activates Nanog, Rex1, and Sox2, which are involved in maintaining the cancer stem cell phenotype and drug resistance [114,116].

CAFs are the most abundant stromal cell population in the EOC microenvironment. They support tumor progression by secreting mitogenic factors, including FGF-1, HGF, VEGF, and PDGF, which promote angiogenesis, tumor cell growth, and invasiveness [115,118]. CAFs also release proangiogenic factors and oxidoreductases that enhance the aggressive phenotype of cancer cells [115,118].

TAMs constitute the predominant immune cell population in the ovarian cancer microenvironment and exhibit substantial functional plasticity, enabling them to shift between an antitumor M1-like phenotype and a protumor M2-like phenotype [119,120,121]. M2-like macrophages predominate within the tumor microenvironment, promoting immunosuppression and disease progression. They contribute to chemoresistance and metastasis by secreting cytokines, chemokines, enzymes, and exosomes, and their presence in tumors is associated with a poorer prognosis [119,120,121].

The ECM plays an important role in shaping EOC growth and metastatic dissemination, as well as in mechanisms of drug resistance. ECM components, including collagen, fibronectin, laminin, and proteoglycans, modulate cell adhesion, integrin signaling, and growth factor bioavailability, thereby creating physical and biochemical barriers that limit drug penetration into the tumor [114,115,122].

#### 3.2.4. Epithelial–Mesenchymal Transition (EMT)

EMT is a key mechanism by which EOC cells acquire an invasive phenotype. This process involves the loss of epithelial markers, particularly E-cadherin, increased expression of mesenchymal markers, including vimentin and N-cadherin, and activation of transcription factors such as ZEB1, SNAIL, and TWIST. In parallel, peritoneal mesothelial cells may undergo mesothelial–mesenchymal transition (MMT), converting them into fibroblast-like cells that support tumor invasion [116,123].

#### 3.2.5. Cellular Senescence

Cellular senescence is a state of permanent cell-cycle arrest in which cells remain metabolically active but lose their capacity to proliferate. It plays a paradoxical role in ovarian cancer biology. On the one hand, senescence serves as a protective mechanism against malignant transformation because senescent cells irreversibly lose their ability to replicate. On the other hand, senescent cells exhibit a characteristic senescence-associated secretory phenotype (SASP), involving the enhanced secretion of cytokines, chemokines, growth factors, and proteases that may promote tumor cell expansion both in vitro and in vivo [124,125,126].

This phenomenon is particularly relevant to ovarian cancer, the progression of which has been shown in cell culture and animal models to be promoted by senescent PMCs [126,127]. Spontaneously senescent primary EOC cells are present in both chemotherapy-naïve and chemotherapy-treated patients, although they are more abundant in the latter group. Importantly, although chemotherapy with carboplatin or paclitaxel induces senescence more strongly, spontaneously senescent EOC cells exhibit greater protumorigenic potential. Their secretome more effectively stimulates the proliferation of non-senescent cancer cells, promotes angiogenesis, and enhances interactions with cells of the tumor microenvironment than the secretome of drug-induced senescent cells. This effect is associated with a distinct SASP profile characterized by increased secretion of VEGF, TGF-β1, IL-6, and HGF [127,128].

Therapy-induced senescence (TIS) is commonly observed following radiotherapy and chemotherapy. Its clinical significance is complex: the beneficial effect arises from the suppression of target-cell proliferation, whereas adverse consequences are driven by the SASP, which may promote disease recurrence and metastatic spread after treatment [124,125,129,130]. Studies have shown that cisplatin-induced senescence in EOC (HGSOC) is associated with a TGF-β-enriched secretome that acts through TGF-β receptor 1 and the AKT/mTOR signaling pathway, thereby promoting protumorigenic phenotypes [129].

Cancer-associated mesothelial cells promote chemoresistance in ovarian cancer through paracrine osteopontin signaling. Osteopontin, the expression of which is markedly increased in the ovarian cancer microenvironment, promotes chemoresistance by activating the CD44 receptor, PI3K/AKT signaling, and ABC transporter activity [117]. Senescent ovarian fibroblasts may also contribute to tumor progression through their secretome, which promotes cancer cell survival, migration, and invasion [130].

#### 3.2.6. Autophagy

Autophagy is a lysosome-dependent catabolic process that degrades damaged organelles and misfolded proteins. It plays a dual role in ovarian cancer biology. During the early stages of carcinogenesis, insufficient autophagy promotes the accumulation of mutations and malignant transformation. In established tumors, however, autophagy activation provides cancer cells with a survival advantage under conditions of metabolic stress and protects them from damage induced by chemotherapy and radiotherapy [131,132,133]. It also plays a key role in cisplatin resistance in EOC. This resistance correlates with activation of the ERK signaling pathway, which promotes autophagy; inhibition of ERK or direct suppression of autophagy sensitizes cancer cells to cisplatin-induced apoptosis [134]. Moreover, autophagy may drive cancer cells into a dormant state, thereby protecting them from the cytotoxic effects of treatment while preserving stem-like properties [131,135].

Lysosomes, as the central organelles of the autophagic pathway, contribute to chemoresistance through drug sequestration, lysosomal exocytosis that enables the efflux of chemotherapeutic agents from cancer cells, selective recycling of cellular components, and disruption of cell death mechanisms, including apoptosis, necroptosis, and ferroptosis [136]. PARP inhibitors induce autophagy as an adaptive resistance mechanism in BRCA-wild-type ovarian cancer cells, and combining PARP inhibitors with autophagy inhibitors such as hydroxychloroquine or chloroquine may improve treatment outcomes [137].

#### 3.2.7. Recurrence and Mechanisms of Tumor Cell Dormancy

EOC recurrence remains one of the greatest challenges in gynecologic oncology. Despite advances in diagnosis and treatment, approximately 70–80% of patients with advanced disease experience relapse within several years after completing first-line therapy [1]. One proposed mechanism is the persistence of cancer stem cells (CSCs), which are characterized by self-renewal and differentiation capacity and reduced sensitivity to chemotherapy, enabling them to survive treatment and subsequently regenerate the tumor [115,122]. The tumor microenvironment supports CSC maintenance through nutrient and oxygen gradients, interactions with the extracellular matrix, immune modulation, and signals derived from cancer-associated fibroblasts [28,31,35]. Dysregulation of WNT, NOTCH, PI3K/AKT/mTOR, TGF-β, JAK/STAT, Hedgehog, NF-κB, and Hippo signaling further promotes CSC stemness, plasticity, and survival [115,122].

As defined in Section 3.2.1, cellular dormancy is distinct from persistent clinically detectable disease and recurrence. This subsection focuses on the mechanisms that allow residual EOC cells to survive treatment in a non-proliferative state and later resume growth. Dormant cells may be less sensitive to chemotherapy because they are not actively dividing [126]. Dormancy-associated mechanisms include stress signals from a hostile tissue microenvironment, altered β1-integrin and EGF signaling, suppression of AKT activity accompanied by increased p130 and p27 expression, reduced urokinase receptor expression, and ARHI-dependent autophagy [126]. Autophagy may support the survival of dormant cells under nutrient-limited conditions and facilitate their subsequent transition from quiescence to renewed proliferation [131,135].

### 3.3. Standard Therapeutic Management of EOC

Surgery is the cornerstone of EOC treatment. According to current guidelines, the objective of optimal cytoreductive surgery is the complete removal of all macroscopic tumor deposits (R0) or the reduction in residual tumor volume to the lowest possible level. When primary cytoreduction is expected to be suboptimal, or there is a high risk of leaving macroscopic residual disease, patients receive neoadjuvant chemotherapy followed by interval cytoreductive surgery. A platinum–taxane regimen, most commonly carboplatin combined with paclitaxel, remains the gold standard for first-line chemotherapy [1].

Given the limited effectiveness of surgery and chemotherapy and the high rate of disease recurrence, there is growing interest in alternative therapeutic strategies. These include molecularly targeted therapies, immunotherapy, modulation of the tumor microenvironment, nanotechnology-based approaches, gene therapy, and anticancer vaccines. Some of these strategies are currently being evaluated in preclinical studies or early-phase clinical trials; however, their therapeutic potential suggests that they may improve treatment outcomes and reduce the risk of recurrence [1].

### 3.4. Molecularly Targeted Therapies

#### 3.4.1. Antiangiogenic Therapies

Angiogenesis is a key process driving EOC progression, enabling tumor growth, vascularization, and the dissemination of cancer cells within the peritoneal cavity. This process is strongly regulated by the VEGF/VEGFR axis, which promotes both endothelial cell proliferation and increased vascular permeability, thereby facilitating ascites formation and the development of a growth factor-rich microenvironment. Consequently, targeting the angiogenic pathway has become an important component of systemic EOC treatment [138,139].

The best-characterized and most widely implemented angiogenesis inhibitor is bevacizumab, a monoclonal antibody directed against VEGF-A. By binding VEGF-A, bevacizumab prevents the activation of VEGFRs on endothelial cells, thereby inhibiting neovascularization, normalizing tumor vasculature, and reducing vascular permeability, which clinically translates into decreased accumulation of peritoneal fluid [27,28].

The process of vascular normalization is important not only for inhibiting angiogenesis but also for improving tumor perfusion, reducing hypoxia, and facilitating the penetration of concurrently administered anticancer agents into tumor tissue [140]. Bevacizumab treatment is associated with prolonged progression-free survival, particularly in patients with advanced or recurrent EOC, as demonstrated in randomized phase III trials [27,28].

In addition to bevacizumab, orally administered tyrosine kinase inhibitors targeting angiogenic pathways, including cediranib and pazopanib, have also been evaluated. Pazopanib is a multikinase inhibitor targeting VEGFR, PDGFR, and FGFR, whereas cediranib selectively inhibits VEGFR-family kinases. Unlike monoclonal antibodies, tyrosine kinase inhibitors act by inhibiting intracellular receptor tyrosine kinases. This distinct mechanism of modulation of angiogenic signaling may result in differences in both efficacy and toxicity profiles [29]. The clinical activity of cediranib has also been investigated in combination with immune checkpoint and PARP inhibition. In a phase I/II, single-center, non-randomized trial in recurrent ovarian cancer, durvalumab plus cediranib achieved an objective response rate of 29.6% and met the prespecified primary endpoint, whereas the addition of olaparib to this combination resulted in an objective response rate of 19.4% and did not meet the primary endpoint. Median progression-free survival was 4.5 months in both treatment groups, although a subset of patients experienced prolonged disease control lasting at least 12 months. Both regimens demonstrated manageable toxicity [30].

Translational analyses further suggested that treatment benefit may depend on the tumor’s molecular characteristics. Baseline tumors from exceptional responders and patients deriving clinical benefit were enriched in immune activation and metabolic pathways, whereas tumors from patients without clinical benefit showed increased activity of pathways associated with vascular adaptation and cytoskeletal remodeling. These findings provide proof of concept for the activity of cediranib-containing combinations and indicate that immune, metabolic, and vascular molecular signatures may have predictive value in selected patients with recurrent ovarian cancer [30].

Despite the long-standing clinical use of bevacizumab, no biomarker has yet to be identified that can reliably predict treatment response. Potential biomarkers investigated to date include VEGF-A concentrations, components of the Tie2/Ang1 axis, IL-6, YKL-40, microvessel density, EGFR/ADAM17 expression, angiogenesis-related microRNAs, and molecular EOC subtypes. However, none of these markers has been validated for routine clinical use [140]. Although initially effective, antiangiogenic therapy frequently leads to the development of resistance. One of the principal mechanisms is the activation of alternative proangiogenic pathways, including FGF, PDGF, and angiopoietin signaling, which compensate for VEGF blockade [31]. Tumors may also adapt to hypoxic conditions by activating HIF-1α, a key transcriptional regulator of the cellular response to oxygen deprivation. This adaptation promotes the acquisition of a more aggressive tumor phenotype characterized by increased invasive and metastatic potential [32].

Cancer cells may also exploit vessel co-option, in which pre-existing blood vessels are incorporated into the tumor vasculature, as well as microenvironmental remodeling involving pericytes and bone marrow-derived cells. These mechanisms maintain tumor vascularization despite effective VEGF inhibition [140]. Remodeling of the tumor microenvironment also plays an important role, including the activation of CAFs and TAMs, which may support angiogenesis through alternative and compensatory signaling pathways, such as the secretion of proangiogenic factors and the modulation of inflammatory responses [33,34].

These adaptive mechanisms indicate that progression during antiangiogenic therapy does not necessarily reflect complete loss of dependence on angiogenic support. Instead, tumors may maintain vascularization through compensatory proangiogenic signaling, vessel co-option, hypoxia-driven adaptation, and microenvironmental remodeling [35].

Prior exposure to bevacizumab does not invariably preclude benefit from subsequent antiangiogenic treatment. In the randomized phase III MITO16B/MaNGO-OV2B/ENGOT-ov17 trial, patients with platinum-sensitive recurrent ovarian cancer previously treated with first-line bevacizumab received a carboplatin-based doublet with or without bevacizumab. Bevacizumab beyond progression improved median progression-free survival compared with chemotherapy alone, supporting retreatment in a selected clinical population [36].

These findings suggest that adaptive resistance to VEGF blockade should not automatically be interpreted as complete clinical cross-resistance. Treatment sequencing should consider platinum sensitivity, the duration and tolerability of previous bevacizumab treatment, the timing and pattern of progression, prior maintenance therapy, residual toxicity, and the risk of gastrointestinal or vascular complications. However, the evidence for bevacizumab retreatment applies specifically to patients with platinum-sensitive recurrence after prior first-line bevacizumab and should not be generalized to all patients who progress during antiangiogenic therapy [36].

In recent years, combination strategies incorporating PARP inhibitors have gained increasing importance, particularly in patients with homologous recombination deficiency (HRD). This rationale is partly based on the observation that tumor microenvironmental hypoxia increases genomic instability and impairs homologous recombination, whereas inhibition of the VEGF pathway may further enhance DNA damage, thereby increasing cancer cell sensitivity to PARP inhibitors [140]. Combining angiogenesis inhibition with disruption of DNA repair produces a synergistic antitumor effect, resulting from both the induction of replication stress and the deterioration of the tumor microenvironment [141].

Preclinical and clinical studies also indicate the potential of combining antiangiogenic therapies with immune checkpoint inhibitors. A translational analysis of the ICON7 trial provided particularly interesting findings, showing that a biomarker panel combining FLT4 (VEGFR-3), α1-acid glycoprotein (AGP), and mesothelin concentrations with CA-125 measurements may improve the identification of patients likely to benefit from bevacizumab treatment. However, these findings require validation in independent cohorts before they can be implemented in clinical practice [140].

The rationale for combining antiangiogenic treatment with immunotherapy is based on vascular normalization, which improves tumor perfusion, reduces hypoxia, and enhances T-cell infiltration into the tumor microenvironment, potentially strengthening antitumor immune responses [142,143].

#### 3.4.2. PARP Inhibitors—A Cornerstone of Targeted Therapy

Poly(ADP-ribose) polymerase inhibitors (PARPis) represent a paradigm of targeted therapy in EOC by exploiting synthetic lethality in cells with homologous recombination deficiency (HRD). PARP1 and PARP2 play key roles in the repair of DNA single-strand breaks (SSBs) through the base excision repair (BER) pathway. PARP inhibition leads to the accumulation of SSBs, which are converted into double-strand breaks (DSBs) during DNA replication. In cells with intact homologous recombination repair (HRR), DSBs are efficiently repaired, whereas in cells harboring BRCA1/2 mutations or other defects causing HRD, the absence of an effective alternative repair pathway results in genomic catastrophe and cell death [1,144].

Three PARP inhibitors have received FDA approval for the treatment of EOC: olaparib, niraparib, and rucaparib [1,145,146,147]. In the SOLO1 trial, first-line maintenance therapy with olaparib was associated with a clinically meaningful improvement in 7-year overall survival compared with placebo in patients with newly diagnosed advanced BRCA1/2-mutated ovarian cancer (67.0% vs. 46.5%; HR, 0.55; 95% CI, 0.40–0.76). However, the prespecified threshold for statistical significance was not reached (*p* = 0.0004; prespecified threshold, *p* < 0.0001) [1,37]. The combination of olaparib and bevacizumab in the PAOLA-1 trial improved median overall survival in patients with HRD-positive tumors (75.2 vs. 57.3 months; HR, 0.62) [1,38,39]. The highest 5-year progression-free survival rate was observed in patients with HRD-positive tumors and lower clinical risk, further confirming the importance of molecular stratification when selecting maintenance therapy [39].

In the PRIMA/ENGOT-OV26 trial, niraparib improved progression-free survival (PFS) regardless of BRCA or HRD status, with the greatest benefit observed in patients with HRD-positive tumors. Despite significant prolongation of PFS reported in numerous PARPi trials, the benefit in overall survival (OS) has not always been unequivocal. Possible explanations include the use of subsequent lines of therapy after progression, heterogeneity of the study populations, development of PARPi resistance, and cumulative toxicity associated with prolonged treatment [145].

Long-term follow-up and updated clinical trial analyses have confirmed that the greatest benefit from PARPi maintenance therapy is achieved in patients with BRCA1/2 mutations or other HRD-related alterations. In patients without these molecular abnormalities, the clinical benefit is considerably smaller, prompting revisions of regulatory indications. Consequently, the US Food and Drug Administration (FDA) has restricted several previously approved PARPi indications, emphasizing the importance of molecular testing and appropriate patient selection before treatment initiation [1,145,147,148].

Mechanisms of PARPi resistance include BRCA1/2 reversion mutations that restore HRR; ABCB1 gene fusions that increase drug efflux; loss of 53BP1 or REV7, which enable alternative DNA repair; and stabilization of replication forks [1,3,40,41,42]. Such resistance is frequently heterogeneous, with several mechanisms potentially coexisting within a single tumor, thereby posing a major therapeutic challenge [3,41].

Several PARPi-resistance mechanisms, particularly restoration of homologous recombination repair and replication-fork stabilization, may also reduce sensitivity to subsequent platinum-based chemotherapy and contribute to cross-resistance. Serial circulating tumor DNA analysis in patients with BRCA1/2-mutated ovarian cancer showed that homologous recombination restoration, replication-fork stabilization, activation of survival pathways, target loss, and drug efflux could coexist after progression during PARPi therapy. Homologous recombination restoration and the presence of multiple resistance mechanisms were associated with poorer outcomes following subsequent chemotherapy [43].

In a post hoc analysis of PAOLA-1/ENGOT-ov25, the efficacy of subsequent therapy varied depending on whether progression occurred during or after first-line olaparib maintenance. The interval from first to second subsequent therapy was shorter in patients who progressed during olaparib treatment, and the timing of progression remained clinically relevant independently of the conventional platinum-free interval [44].

These findings indicate that the interval from the last platinum dose should not be interpreted in isolation after PARPi exposure. Treatment sequencing should also consider whether progression occurred during or after PARPi maintenance, the depth and duration of the previous platinum response, BRCA/HRD status, and the potential emergence of molecular mechanisms shared between PARPis and platinum resistance [43,44].

Long-term PARPi therapy is also associated with the risk of cumulative adverse events, particularly myelosuppression and, less commonly, myelodysplastic syndromes (MDSs) and acute myeloid leukemia (AML) [145]. This risk increases with treatment duration and prior exposure to platinum-based chemotherapy, underscoring the need for long-term safety monitoring [145].

#### 3.4.3. Antibody–Drug Conjugates

Antibody–drug conjugates (ADCs) are among the most rapidly evolving classes of therapeutics in gynecologic oncology. ADCs combine the targeting precision of a monoclonal antibody with the potency of a cytotoxic payload, thereby delivering a highly active agent directly to tumor cells expressing a specific antigen. The modular structure of an ADC—comprising an antibody, a linker, and a cytotoxic payload—enables the optimization of each component to enhance therapeutic efficacy while reducing systemic toxicity [53,149].

Mirvetuximab soravtansine (MIRV) is the first ADC approved for EOC and targets folate receptor alpha (FRα). MIRV consists of an anti-FRα antibody, a cleavable linker, and the maytansinoid DM4, a potent tubulin inhibitor. FRα is commonly overexpressed in EOC cells, particularly in HGSOC, and is minimally expressed in normal tissues, making it an attractive therapeutic target [45].

In the phase III MIRASOL trial (*n* = 453), MIRV demonstrated statistically significant superiority over chemotherapy in patients with platinum-resistant HGSOC and high FRα expression, defined as ≥75% of tumor cells with staining intensity ≥ 2+. Median PFS was 5.62 versus 3.98 months (*p* = 0.001), ORR was 42.3% versus 15.9% (*p* = 0.001), and median OS was 16.46 versus 12.75 months (HR, 0.67; *p* = 0.005) [45].

The safety profile of MIRV was more favorable than that of chemotherapy, with fewer grade ≥ 3 adverse events (41.7% vs. 54.1%), fewer serious adverse events (23.9% vs. 32.9%), and fewer treatment discontinuations (9.2% vs. 15.9%). The most common adverse events included ocular toxicities, such as blurred vision and keratopathy, as well as gastrointestinal symptoms [45,46].

The combination of mirvetuximab soravtansine and bevacizumab achieved an ORR of 44%, a median duration of response of 9.7 months, and a median PFS of 8.2 months in patients with FRα expression in ≥25% of tumor cells at a staining intensity of ≥2+. Antitumor activity was observed irrespective of the level of FRα expression [47].

Trastuzumab deruxtecan (T-DXd) is an ADC that targets HER2 (ERBB2). In 2024, the FDA granted tumor-agnostic accelerated approval for T-DXd in adults with unresectable or metastatic HER2-positive solid tumors, defined as HER2 IHC 3+, who had received prior systemic treatment and had no satisfactory alternative treatment options. This indication may include selected patients with EOC meeting these criteria, but it does not constitute an EOC-specific approval. T-DXd consists of an anti-HER2 antibody, a cleavable tetrapeptide linker, and a topoisomerase I inhibitor payload (DXd). Its bystander effect may enable cytotoxic activity against neighboring tumor cells with lower or heterogeneous HER2 expression [1,150]. ADCs currently under clinical investigation include sacituzumab govitecan, which targets TROP-2; upifitamab rilsodotin, which targets NaPi2b; and ADCs directed against mesothelin, cadherin-6, B7-H4, and claudin-6.

Major challenges associated with ADC therapy include heterogeneous antigen expression, treatment-related toxicities, and acquired resistance [48,149]. Target expression may differ among histologic subtypes, individual tumor deposits, and successive stages of disease. A recent analysis of paired and longitudinal EOC specimens demonstrated histotype-specific expression patterns and a directional reduction in the cognate target after ADC exposure, particularly for FOLR1, HER2, and CDH6. Although these findings were based on a limited number of paired post-treatment samples, they suggest that antigen expression may evolve under therapeutic pressure and that archival tissue may not always fully represent the target profile at the time of subsequent ADC selection [49].

Earlier paired analyses of archival and fresh biopsy specimens showed 71% concordance of FRα expression and no major shifts between matched pre- and post-mirvetuximab samples. These findings support the practical use of archival tissue in many patients, but also demonstrate that discordance can occur and that fresh tissue may be informative when the archival specimen is old, inadequate, or inconsistent with the current clinical course [50].

Repeat tissue biopsy could therefore help reassess ADC-target expression at recurrence or after prior target-directed therapy, particularly when treatment eligibility depends on a defined immunohistochemical threshold. However, a single biopsy may not capture spatial heterogeneity among multiple metastatic sites and may be limited by procedural feasibility, sampling error, and insufficient viable tumor tissue [49,50].

Liquid biopsy represents a less invasive potential method for longitudinal target assessment, but its role in ADC selection remains investigational. Detection of ADC-target expression on circulating tumor cells from patients with EOC appears feasible and may provide real-time information on target status. However, prospective validation and assay standardization are required, and circulating cells must be shown to represent target expression across clinically relevant tumor deposits before this method can replace tissue-based assessment [51].

Acquired ADC resistance may result from antigen loss or downregulation, impaired internalization or intracellular trafficking, altered payload release, payload-specific resistance, increased drug efflux, and impaired apoptotic signaling. Together with temporal and spatial heterogeneity in targets, these mechanisms support longitudinal biomarker reassessment and careful sequencing of ADCs with different targets or payloads, rather than assuming persistent sensitivity based on a single historical biopsy [52].

#### 3.4.4. Replication Stress Pathway Inhibitors: WEE1 and ATR

Replication stress is a therapeutic vulnerability in cancer cells due to their high proliferative rate and the loss of normal mechanisms that repair DNA and prevent replication errors. ATR (ataxia telangiectasia and Rad3-related protein) and WEE1 are key regulators of the cellular DNA damage response and are frequently overexpressed in cancer cells, thereby increasing their tolerance to replication stress [53,151].

Adavosertib (AZD1775), a first-generation WEE1 inhibitor, demonstrated clinical activity in phase II trials. In combination with carboplatin, it achieved an objective response rate (ORR) of 38–67% and a disease control rate (DCR) of up to 100%. In patients with platinum-resistant EOC, the combination of adavosertib and gemcitabine prolonged progression-free survival (median, 4.6 vs. 3.0 months) and overall survival (median, 11.4 vs. 7.2 months) compared with gemcitabine monotherapy [53,54]. Despite these encouraging results, the clinical development of adavosertib was discontinued because of hematologic toxicity. The second-generation inhibitor ZN-c3 achieved a DCR of 80% in a phase I trial and demonstrated an acceptable safety profile [53].

Berzosertib, a first-in-class ATR inhibitor, was evaluated in combination with gemcitabine in a randomized phase II trial involving patients with platinum-resistant high-grade serous ovarian cancer. In the overall study population, the combination improved median progression-free survival compared with gemcitabine alone (22.9 vs. 14.7 weeks; HR, 0.57; 90% CI, 0.33–0.98; one-sided *p* = 0.044) [54]. In the final analysis, median overall survival was 59.4 versus 43.0 weeks (HR, 0.79; 90% CI, 0.52–1.20; one-sided *p* = 0.18), and therefore no statistically significant overall-survival benefit was demonstrated in the overall population. A possible OS benefit was observed in an exploratory subgroup with a platinum-free interval of ≤3 months, but this analysis included only 26 patients and requires prospective validation [152].

#### 3.4.5. PI3K/AKT/mTOR Pathway Inhibition

The PI3K/AKT/mTOR pathway is one of the most frequently dysregulated signaling pathways in EOC and plays a key role in cell proliferation, survival, metabolism, and treatment resistance. Activation of this pathway promotes cancer cell survival and contributes to chemoresistance [153,154,155].

Despite a strong biological rationale, clinical trials evaluating PI3K/AKT/mTOR inhibitors as monotherapies have yet to demonstrate meaningful clinical benefit. This reflects the pathway’s complexity and highlights the need for combination strategies [154,155]. To date, no PI3K, AKT, or mTOR inhibitor has been approved for the treatment of EOC, while the development of dual PI3K/mTOR inhibitors has been substantially limited by safety concerns and toxicities observed in early-phase clinical trials [156].

AKT inhibitors, including afuresertib, MK-2206, and AZD5363/capivasertib, have shown promising but variable antitumor activity in preclinical and clinical studies [53,153]. Particularly encouraging results have been reported for combinations of PI3K/AKT/mTOR pathway inhibitors with PARP inhibitors. Such combinations may overcome PARPi resistance in homologous recombination-proficient (HRP) tumors by synergistically inhibiting DNA repair and prosurvival signaling [154,155].

The combination of serabelisib, a PI3Kα inhibitor, and sapanisertib, an mTOR inhibitor, with paclitaxel—the PIKTOR regimen—achieved an ORR of 47% in a phase I trial involving patients with advanced ovarian, endometrial, and breast tumors that had progressed during taxane treatment [157].

#### 3.4.6. MAPK Pathway Inhibitors

The RAS–MEK–ERK pathway is a key regulator of cell proliferation, differentiation, and survival. Activating mutations in KRAS (up to 54%), BRAF (15–16%), and NRAS (12–13%) are characteristic of low-grade serous carcinoma (LGSOC), making the MAPK pathway a major therapeutic target in this subtype [55,56].

Trametinib, a MEK1/2 inhibitor, demonstrated superiority over the physician’s choice standard-of-care treatment, including chemotherapy or endocrine therapy, in the randomized phase II/III GOG 281/LOGS trial involving patients with recurrent LGSOC. Median PFS was 13.0 months with trametinib versus 7.2 months with standard therapy (HR, 0.48; 95% CI, 0.36–0.64; *p* < 0.0001) [56]. In contrast to LGSOC, MEK inhibitors have not yielded comparable clinical benefits in high-grade serous carcinoma (HGSOC), highlighting biological differences between these EOC subtypes [56].

Avutometinib in combination with defactinib received accelerated FDA approval in 2025 for adults with KRAS-mutant recurrent LGSOC who had previously received systemic therapy [158]. Because the approval was accelerated, confirmatory randomized evidence is still required to verify clinical benefit. In contrast, binimetinib did not improve PFS compared with the physician’s choice chemotherapy in the phase III MILO/ENGOT-ov11 trial. Median PFS was 9.1 months with binimetinib and 10.6 months with chemotherapy (HR, 1.21; 95% CI, 0.79–1.86), and the study was closed early after crossing the prespecified futility boundary [57]. Post hoc analyses showed higher response rates and longer progression-free survival among binimetinib-treated tumors harboring MAPK pathway alterations than among those without such alterations; however, these findings remain exploratory and have not established a prospectively validated biomarker-based indication for binimetinib [58].

These discordant findings indicate that inhibition of the MAPK pathway cannot be considered a uniform class effect in LGSOC. Clinical activity depends on the specific agent, trial population, molecular context, and study design. At present, trametinib has randomized comparative evidence, whereas avutometinib plus defactinib has accelerated regulatory approval for KRAS-mutated recurrent LGSOC, and the role of binimetinib remains unestablished.

According to the NCCN Guidelines, version 2.2026, binimetinib is listed as a category 2B treatment option for patients with recurrent LGSOC. In the same clinical setting, avutometinib combined with defactinib has demonstrated clinically meaningful and durable activity, with a confirmed overall objective response rate of 31% and greater activity in KRAS-mutant tumors, supporting its use as a potential treatment option for recurrent LGSOC [159,160].

#### 3.4.7. Glucocorticoid Receptor Antagonists

Relacorilant, a first-in-class selective glucocorticoid receptor (GR) antagonist, represents a novel therapeutic approach that modulates cortisol signaling within the tumor microenvironment. In ovarian cancer, GR signaling contributes to reduced tumor sensitivity to chemotherapy, while increased GR expression has been associated with a poorer prognosis [161].

In the randomized, open-label phase III ROSELLA trial (GOG-3073/ENGOT-ov72; n = 381), patients with platinum-resistant ovarian, fallopian tube, or primary peritoneal cancer who had received one to three prior systemic treatment regimens were assigned to relacorilant plus nab-paclitaxel or nab-paclitaxel monotherapy. The combination significantly prolonged progression-free survival compared with nab-paclitaxel alone (median, 6.54 vs. 5.52 months; HR, 0.70; 95% CI, 0.54–0.91; *p* = 0.0076) [161].

In the final overall-survival analysis, relacorilant plus nab-paclitaxel also improved median overall survival (16.0 vs. 11.9 months; HR, 0.65; 95% CI, 0.51–0.83; *p* = 0.0004). The 18-month overall-survival rates were 46% and 27%, respectively. Treatment-related adverse events remained generally consistent with the known safety profile of the component therapy, and no new safety signals were identified with longer follow-up [162].

On 25 March 2026, the FDA approved relacorilant (Lifyorli^®^) in combination with nab-paclitaxel for adults with platinum-resistant epithelial ovarian, fallopian tube, or primary peritoneal cancer who had received one to three prior systemic treatment regimens, at least one of which included bevacizumab [163]. The approval did not require selection based on a molecular biomarker.

#### 3.4.8. Tumor-Agnostic Therapies

FDA-approved tumor-agnostic targeted therapies are used for solid tumors harboring specific molecular alterations, regardless of primary tumor site. These include NTRK inhibitors such as larotrectinib, entrectinib, and repotrectinib; the RET inhibitor selpercatinib; BRAF V600E-targeted therapy with dabrafenib plus trametinib; and pembrolizumab immunotherapy for tumors with microsatellite instability-high or mismatch repair-deficient status (MSI-H/dMMR) or high tumor mutational burden (TMB-H) [164].

#### 3.4.9. Immunotherapy

EOC is immunologically classified as a “cold tumor,” characterized by low T-cell infiltration, limited neoantigen expression, and a strongly immunosuppressive tumor microenvironment [60,61]. Nevertheless, the presence of tumor-infiltrating lymphocytes (TILs), particularly CD8+ T cells, is associated with a more favorable prognosis, indicating the potential value of immunotherapy in appropriately selected patient subgroups [61,165].

Mechanisms of immunosuppression in EOC include the expression of immune checkpoint ligands such as PD-L1 and B7-H4, recruitment of regulatory T cells (Tregs) and myeloid-derived suppressor cells (MDSCs), polarization of macrophages toward the M2 phenotype, secretion of immunosuppressive cytokines including TGF-β, IL-10, and VEGF, and metabolic competition for nutrients within the tumor microenvironment [60,61,165].

Despite their transformative impact on the treatment of many solid tumors, immune checkpoint inhibitors have shown limited efficacy as monotherapy in EOC. Response rates to anti-PD-1/PD-L1 monotherapy range from only 8% to 22% [61,62,63]. Several phase III trials have yielded negative results. In JAVELIN Ovarian 100, avelumab, an anti-PD-L1 antibody, administered either in combination with chemotherapy or as maintenance therapy in patients with previously untreated EOC, did not improve progression-free survival (PFS) compared with chemotherapy alone. The trial was discontinued at the interim analysis after meeting the predefined futility criteria [64].

In IMagyn050, the addition of atezolizumab, an anti-PD-L1 antibody, to chemotherapy and bevacizumab in patients with newly diagnosed advanced EOC did not significantly improve PFS in either the overall population or the PD-L1-positive subgroup [64]. Similarly, in JAVELIN Ovarian 200, avelumab administered alone or in combination with pegylated liposomal doxorubicin failed to improve overall survival (OS) or PFS compared with pegylated liposomal doxorubicin alone in patients with platinum-resistant EOC [53,65].

Nevertheless, several approaches have shown promising activity. Dual immune checkpoint blockade with nivolumab plus ipilimumab produced a higher response rate than nivolumab monotherapy in platinum-resistant EOC (31% vs. 12%), although no improvement in OS was observed [53]. In the phase III DUO-O/ENGOT-ov46 trial, patients without tumor BRCA mutations received durvalumab in combination with first-line carboplatin, paclitaxel, and bevacizumab, followed by maintenance durvalumab, bevacizumab, and olaparib. Compared with the control regimen of chemotherapy plus bevacizumab followed by bevacizumab maintenance, the experimental regimen improved PFS in the non-tBRCAm intention-to-treat population (median, 24.2 vs. 19.3 months; HR, 0.63; 95% CI, 0.52–0.76) and produced a greater relative benefit in the non-tBRCAm HRD-positive population (median, 37.3 vs. 23.0 months; HR, 0.49; 95% CI, 0.34–0.69). At the interim OS analysis, no statistically significant difference was observed in the non-tBRCAm intention-to-treat population (HR, 0.95; 95% CI, 0.76–1.20; *p* = 0.68), with 39.0% data maturity. [166].

In KEYLYNK-001, maintenance therapy with pembrolizumab plus olaparib improved PFS in patients with a combined positive score (CPS) of ≥10 (median PFS, 23.7 vs. 15.2 months; HR, 0.63; *p* = 0.0001) [145]. In the phase III FIRST/ENGOT-OV44 trial, the addition of dostarlimab to first-line platinum-based chemotherapy, followed by maintenance therapy with dostarlimab plus niraparib, resulted in a statistically significant but clinically modest improvement in progression-free survival compared with chemotherapy followed by niraparib maintenance alone (median, 20.6 vs. 19.2 months; HR, 0.85; 95% CI, 0.73–0.99; *p* = 0.0351). No overall-survival improvement was observed (median, 44.4 vs. 45.4 months; HR, 1.01; 95% CI, 0.86–1.19; *p* = 0.9060) [167].

#### 3.4.10. Biomarker-Based Strategies

PD-L1 and CD8 expression are being investigated as potential predictors of response to immune checkpoint inhibitors. A favorable trend in objective response rate (ORR) was observed in patients with high PD-L1 and/or CD8 expression treated with avelumab in JAVELIN Ovarian 200 or pembrolizumab in KEYNOTE-100. However, the findings were inconsistent across studies, potentially due to differences in the diagnostic assays used to assess PD-L1 expression [53].

Current NCCN recommendations (version 3.2026) for immunotherapy in EOC include dostarlimab for recurrent or advanced dMMR/MSI-H tumors; pembrolizumab for MSI-H/dMMR or tumor mutational burden-high tumors (TMB-H; ≥10 mutations per megabase); paclitaxel plus pembrolizumab, with or without bevacizumab, for PD-L1-positive tumors (CPS ≥1) in platinum-resistant disease; and ipilimumab plus nivolumab specifically for platinum-resistant clear cell carcinoma [59].

### 3.5. Adoptive Cell Therapies

#### 3.5.1. Chimeric Antigen Receptor T-Cell Therapy

Chimeric antigen receptor T-cell (CAR-T) therapy is an active area of research in EOC, although its application in solid tumors faces substantial challenges compared with hematologic malignancies [66,67,68]. The principal target antigens investigated in EOC include mesothelin (MSLN), MUC16/CA-125, folate receptor alpha (FRα), HER2, and B7-H3.

MSLN is overexpressed in more than 70% of serous EOCs and is among the most extensively investigated CAR-T targets in this disease. In orthotopic models, MSLN-targeted CAR-T cells containing a CD28 costimulatory domain prolonged survival but failed to provide durable tumor control, whereas constructs incorporating a 4-1BB domain induced long-term remission in a subset of mice [53,69]. MUC16 also represents an attractive target. K101CAR-T cells directed against the extracellular repeat domain of CA-125/MUC16 demonstrated specific cytotoxicity against ovarian cancer cells in vitro and in vivo, including in orthotopic patient-derived xenograft models, and their activity was not inhibited by soluble CA-125 [70]. Fully human CAR-T cells targeting FOLR1 selectively recognized and eliminated FRα-expressing EOC cells in preclinical models [53,71]. HER2-directed CAR-T cells also suppressed tumor growth, with intraperitoneal administration providing more effective local control than intravenous delivery in the evaluated model [72]. B7-H3-directed CAR-T cells demonstrated potent antitumor activity and reduced features of CAR-T-cell exhaustion in preclinical EOC models [73].

Strategies designed to address antigen heterogeneity and limited CAR-T-cell persistence include dual-target and alternative signaling constructs. B4M3 CAR-T cells targeting both MSLN and B7-H3 inhibited tumor growth, prolonged survival, increased cytotoxic T-cell infiltration, and reduced regulatory T-cell abundance in xenograft models [74,75]. Mesothelin-targeted engineered T cells based on KIR/DAP12 signaling were developed to limit T-cell exhaustion and improve response durability. Preclinical studies and the first clinical trial involving mesothelin-expressing malignancies, including EOC, demonstrated a favorable safety profile and preliminary antitumor activity [76]. Bispecific CAR-T cells simultaneously targeting CD155 and ROR1 showed greater cytotoxicity, reduced tonic signaling, and delayed exhaustion compared with single-target constructs [77].

Despite encouraging preclinical and early clinical findings, CAR-T therapy in EOC remains limited by several major barriers. These include heterogeneous tumor antigen expression, limited infiltration, and long-term persistence of CAR-T cells in solid tumors; an immunosuppressive microenvironment containing myeloid-derived suppressor cells, regulatory T cells, tumor-associated macrophages, TGF-β, and IL-10; and physical barriers such as ascites and a dense extracellular matrix. Cytokine release syndrome and neurotoxicity represent potential safety concerns [66,67,68].

Approaches under investigation to overcome these limitations include intraperitoneal administration; CRISPR-based modifications intended to reduce alloreactivity and T-cell exhaustion; constructs expressing cytokines such as IL-15 or IL-21; combinations with immune checkpoint inhibitors; and dual- or multitarget CAR-T strategies designed to improve the elimination of heterogeneous tumor cell populations [66,67,68].

#### 3.5.2. CAR-NK Therapy

Chimeric antigen receptor natural killer (CAR-NK) cells represent a promising alternative to CAR-T therapy. Compared with CAR-T cells, CAR-NK cells are associated with a lower risk of cytokine release syndrome (CRS) and neurotoxicity, can be used in allogeneic settings without HLA matching, and retain their innate ability to recognize and eliminate tumor cells independently of CAR expression. In addition, their shorter persistence may reduce the risk of prolonged off-target toxicity. In preclinical EOC models, CAR-NK cells targeting mesothelin, HER2, and NKG2D have demonstrated antitumor activity; however, their clinical efficacy in EOC remains unestablished [66].

#### 3.5.3. Therapeutic Cancer Vaccines

Therapeutic cancer vaccines are designed to induce or enhance antitumor immune responses by presenting tumor-associated antigens to the immune system. Several vaccine platforms are currently being investigated in EOC, all of which aim to activate antigen-specific T-cell responses against tumor cells.

Dendritic cell (DC)-based vaccines use DCs loaded with tumor antigens, such as tumor lysates, peptides, or mRNA. Following administration, these cells present antigens to the immune system, initiating a T-cell-mediated response. Peptide-based vaccines consist of synthetic peptides corresponding to epitopes derived from selected tumor antigens, including NY-ESO-1, WT1, p53, and HER2. Neoantigen vaccines represent a personalized approach based on patient-specific tumor neoantigens identified through genomic sequencing [62,63,165].

#### 3.5.4. Oncolytic Viruses

Oncolytic viruses selectively infect and destroy tumor cells while simultaneously stimulating antitumor immune responses by releasing tumor antigens and danger-associated molecular patterns (DAMPs). Modified adenoviruses, herpes simplex viruses (HSVs), reoviruses, and Newcastle disease viruses are among the platforms currently being investigated in EOC [165].

#### 3.5.5. Engineered Cytokines and Bispecific Antibodies

Nemvaleukin alfa is an engineered cytokine that selectively engages IL-2Rβ to activate CD8+ T cells and natural killer (NK) cells. In an early-phase trial involving patients with platinum-resistant EOC, it achieved an objective response rate (ORR) of 28.6% and a disease control rate (DCR) of 71.4%. The phase III ARTISTRY-7 trial is evaluating nemvaleukin alfa in combination with pembrolizumab [53,168].

Bispecific antibodies are constructs containing two distinct antigen-binding sites, enabling simultaneous targeting of a tumor-associated antigen and a T-cell receptor, such as CD3. They are being investigated as a strategy to enhance antitumor immune responses in solid tumors, including ovarian cancer [165].

Overall, the clinical evidence supporting CAR-NK therapy, therapeutic cancer vaccines, oncolytic viruses, engineered cytokines, and bispecific antibodies in EOC remains limited. CAR-NK data in EOC are derived predominantly from preclinical models, and clinical efficacy, optimal antigen selection, persistence, manufacturing, and delivery strategies remain to be established [66]. Most ovarian cancer vaccine studies have not progressed beyond phase I–II development; although antigen-specific immune responses have frequently been demonstrated, these have not consistently translated into objective clinical benefit [169]. Oncolytic virus studies in EOC are likewise dominated by preclinical and early-phase clinical investigations, with generally acceptable safety profiles but limited evidence of durable survival benefit [170]. Evidence for engineered cytokines and bispecific antibodies is also based mainly on early-phase studies or trials involving heterogeneous tumor populations, and standardized biomarker-based selection has yet to be established [53,171].

Across these approaches, major barriers include heterogeneous or unstable antigen expression, inadequate trafficking and persistence of immune effector cells, and immunosuppression within ascites and the peritoneal tumor microenvironment. Additional challenges include T-cell or NK-cell dysfunction, antiviral immune responses, and platform-specific systemic toxicities [66,165,170,171]. Accordingly, mechanistic activity, immunogenicity, or early response signals should not be interpreted as evidence of established clinical benefit. Further development will require EOC-specific prospective trials, validated biomarker-guided patient selection, standardized manufacturing and delivery platforms, and comparative evaluation against currently available treatments.

### 3.6. Metabolic Therapies

#### 3.6.1. Metabolic Reprogramming as a Hallmark of EOC

Metabolic reprogramming is a key hallmark of EOC that enables tumor cells to survive therapeutic stress, evade immune responses, and develop treatment resistance [1,2]. EOC cells can dynamically switch among glycolysis, oxidative phosphorylation (OXPHOS), glutaminolysis, and lipid metabolism in response to substrate availability, microenvironmental conditions, and treatment pressure. This metabolic plasticity represents a major barrier to therapies directed against a single metabolic pathway [172,173,174].

Metabolic dependencies differ among histologic subtypes. HGSOC frequently exhibits increased reliance on OXPHOS and lipid metabolism, reflecting its adaptation to the lipid-rich environment of the peritoneal cavity and greater omentum [78,175]. By contrast, clear cell carcinoma is characterized by enhanced aerobic glycolysis and glycogen accumulation, frequently associated with ARID1A and PIK3CA alterations [78,172].

The principal dysregulated pathways in EOC include glycolysis, OXPHOS, glutaminolysis, lipid metabolism, and one-carbon metabolism [78,172,176]. Increased glycolysis is associated with advanced disease, chemoresistance, and poorer prognosis [79,80,81,177,178], whereas OXPHOS is particularly important for the metabolism of HGSOC and cancer stem cells. Lysophosphatidic acid may further promote glycolysis through ROS-dependent stabilization of HIF-1α and induction of GLUT1 and HKII expression [82].

#### 3.6.2. Targeting Glycolysis and Oxidative Phosphorylation

Targeting glycolysis or oxidative phosphorylation (OXPHOS) alone may be insufficient because EOC cells can compensate by switching between metabolic pathways. STF31, a GLUT1 inhibitor, reduced glucose uptake, whereas its combination with the OXPHOS inhibitor metformin synergistically suppressed ovarian cancer cell growth, supporting the simultaneous blockade of glycolysis and mitochondrial metabolism as a strategy to overcome metabolic plasticity [177]. Hexokinase inhibitors, including 3-bromopyruvate and 2-deoxyglucose, induced apoptosis and G1 cell-cycle arrest, and 3-bromopyruvate inhibited tumor growth and prolonged survival in an ovarian cancer xenograft model [82,83]. The PFKFB3 inhibitor 3PO also enhanced the effects of cisplatin and paclitaxel in platinum-sensitive and platinum-resistant cells [177].

Dichloroacetate redirects tumor-cell metabolism toward OXPHOS and may increase susceptibility to oxidative stress and chemotherapy. However, the relatively high concentrations required for antitumor activity limit its potential as monotherapy, shifting interest toward combinations with chemotherapy or metabolic agents such as metformin [84,178]. Cryptotanshinone simultaneously inhibited glycolysis and OXPHOS by reducing glucose uptake, lactate production, and the activities of LDHA and HK2, and by impairing mitochondrial complex I function, thereby inhibiting tumor growth in an EOC xenograft model [179].

Combining glycolytic inhibitors with OXPHOS inhibitors, such as metformin, or glutaminolysis inhibitors, such as aminooxyacetate, has shown synergistic activity by limiting compensatory metabolic switching [83,177]. However, the clinical translation of these strategies is constrained by metabolic plasticity and intratumoral metabolic heterogeneity, which may permit tumor cells to use alternative nutrients and energy-producing pathways. The context-dependent nature of metabolic dependencies also complicates patient selection and supports the need for reliable biomarkers and rational combination strategies [85].

HGSOC frequently depends on mitochondrial metabolism, making OXPHOS a potential therapeutic target in treatment-resistant disease [175]. Investigated approaches include the complex I inhibitor IACS-010759, the complex III inhibitor atovaquone, and the biguanides metformin and phenformin. These agents suppress mitochondrial respiration and energy production, although most evidence in EOC remains preclinical [86].

#### 3.6.3. Targeting Glutamine Metabolism

Glutamine provides ovarian cancer cells with substrates for nucleotide, amino acid, and glutathione synthesis and replenishes the tricarboxylic acid cycle through anaplerotic reactions [78,87,88]. Increased glutaminase 1 (GLS1) activity is associated with elevated glutathione levels and intrinsic resistance to platinum-based chemotherapy, supporting tumor-cell survival under oxidative and therapeutic stress [89].

Glutamine dependence may arise from distinct molecular alterations. MYC amplification or overexpression is associated with chemotherapy resistance and increased GLS1 transcription, identifying the MYC–GLS1 axis as a potential therapeutic vulnerability [89]. In clear cell carcinoma, ARID1A loss relieves repression of GLS1, increasing glutamine utilization in the tricarboxylic acid cycle and aspartate synthesis [180]. Platinum-resistant EOC cells may also increase the expression of the glutamine transporter ASCT2 and glutaminase [88,91]. ASCT2 overexpression is associated with mTOR activation, greater tumor aggressiveness, and shorter survival, whereas its inhibition suppresses proliferation, disrupts redox homeostasis, and increases treatment sensitivity [88,91,92].

GLS1 inhibitors have therefore been investigated as a means of targeting glutamine-dependent resistance. BPTES enhanced chemotherapy-induced growth inhibition and apoptosis in platinum-sensitive and platinum-resistant EOC cells [91,93]. Its activity results from stabilization of an inactive GLS1 conformation, although limited bioavailability has encouraged the development of additional inhibitors [88,94]. These include Compound 968 and the orally administered inhibitor CB-839 (telaglenastat), which has progressed to clinical evaluation [94].

In a phase I trial involving 120 patients with solid tumors, CB-839 produced substantial inhibition of platelet and intratumoral GLS activity and an overall disease-control rate of 43%; however, the reported responses were mainly observed outside EOC [95]. In preclinical EOC models, CB-839, combined with metformin, selectively inhibited KRAS-mutant tumors characterized by increased glutamine metabolism [181]. The oral GLS1 inhibitor IACS-6274 was also evaluated in a phase I trial involving eight patients with EOC. Durable disease stabilization was reported in tumors with ASNS loss, although these findings remain limited to conference proceedings [182].

Glutaminase inhibition may also affect the tumor immune microenvironment. Compound 968 combined with PD-L1 blockade enhanced CD8+ T-cell granzyme B secretion, tumor-cell apoptosis, and intratumoral CD3+ T-cell infiltration and prolonged survival in murine EOC models [183]. More broadly, inhibition of glutaminolysis may restrict glutamine availability to tumor cells while supporting T-cell effector function, providing a rationale for combinations with immunotherapy and PARP inhibitors [84].

The effectiveness of glutaminase inhibition depends on the degree of tumor reliance on glutamine, underscoring the need for biomarkers that identify patients most likely to benefit [88,91]. Clinical translation is further complicated by metabolic heterogeneity and plasticity, context-dependent glutamine dependence, compensatory use of alternative nutrients, and the dual effects of glutamine restriction on tumor and immune cells. These barriers support biomarker-guided patient selection and rational combination strategies rather than the unselected use of glutamine-metabolism inhibitors [94].

#### 3.6.4. Arginine Metabolism-Targeted Therapies

Arginine supports the synthesis of nitric oxide, polyamines, and other metabolites involved in cancer cell proliferation and survival. EOC cells may exhibit increased arginine demand, while overexpression of the arginine transporter SLC7A1/CAT-1 is associated with enhanced proliferation, migration, and poorer clinical outcomes [184,185,186,187].

Pharmacologic arginine depletion represents a potential therapeutic strategy. In preclinical EOC models, pegylated human arginase HuArgI(Co)-PEG5000 inhibited tumor-cell proliferation and invasiveness by inducing autophagy and modulating Rho-family GTPase activity [188]. However, treatment efficacy depends on the capacity of tumor cells to synthesize arginine endogenously. Consequently, variable expression or re-expression of arginine-biosynthetic enzymes may permit metabolic adaptation and resistance, emphasizing the need for predictive biomarkers of arginine auxotrophy.

Clinical translation is also complicated by the broader role of arginine metabolism in the tumor microenvironment. Arginine availability influences not only malignant cells but also antitumor T cells, myeloid-derived suppressor cells, and macrophages. Therefore, systemic arginine depletion may produce context-dependent effects on both tumor metabolism and immune function. These shared barriers support biomarker-guided patient selection and careful evaluation of rational combination strategies [189].

#### 3.6.5. Targeting Lipid Metabolism

Lipid metabolism supports EOC growth and dissemination within the lipid-rich environment of the greater omentum and peritoneal adipose tissue. EOC cells increase both de novo fatty acid synthesis and fatty acid β-oxidation (FAO), providing substrates for membrane formation, signaling, and energy production while limiting lipotoxic stress [190,191]. Exogenous lipid uptake is mediated in part by CD36 and fatty acid-binding protein 4 (FABP4), which facilitate metabolic interactions between EOC cells and omental adipocytes and support peritoneal metastasis [190,192].

Several enzymes involved in lipid synthesis and oxidation have been investigated as therapeutic targets. FASN, ACC, ACLY, and SCD1 contribute to de novo lipid synthesis and reduce tumor dependence on exogenous fatty acids [190,193]. CPT1A, the rate-limiting enzyme of FAO, is overexpressed in EOC and is associated with poorer prognosis and resistance to anoikis. Its expression may be increased by tumor microenvironment-derived TGF-β through AMPK- and NRF2-dependent signaling [194,195]. The CPT1 inhibitor etomoxir suppressed FAO and inhibited tumor growth in preclinical EOC ovarian cancer models, including patient-derived xenografts [195].

Additional metabolic regulators include ACAD9, ACC2, and the DLAT/JAK2/STAT5/SREBP1 axis. ACAD9 has recently been implicated in HGSOC metabolic adaptation, supporting electron-transport-chain integrity and linoleic acid metabolism, whereas its loss induces metabolic collapse and ferroptosis [196]. Reduced ACC2 expression may enhance FAO through dysregulation of the MARCH 5–ACC2 axis [197], whereas DLAT silencing decreases intracellular lipid content and inhibits EOC cell proliferation, migration, and invasion [198].

Cholesterol metabolism also represents a potential therapeutic target. Statins inhibit HMG-CoA reductase and reduce the synthesis of isoprenoids required for protein prenylation. Lipophilic statins, including simvastatin and atorvastatin, have demonstrated preclinical antitumor activity in EOC, although their combination with PD-1 blockade requires clinical validation [199]. SOAT1 regulates cholesterol esterification and storage and is overexpressed in EOC [200]. The SOAT1 inhibitor avasimibe suppressed tumor-cell growth in preclinical models and has been investigated in combination with immune-modulating strategies, but its use in EOC remains preclinical [201].

Despite the number of potentially targetable enzymes, the clinical translation of lipid metabolism inhibitors is constrained by pathway redundancy and metabolic plasticity. Tumor cells may compensate for inhibition of lipid synthesis by increasing exogenous lipid uptake or FAO, while inhibition of one lipid pathway may alter other interconnected metabolic processes. Lipid availability also varies with diet, tissue location, and the tumor microenvironment, complicating the identification of stable dependencies and predictive biomarkers. Moreover, lipid metabolism is essential in normal tissues and immune cells, creating the potential for systemic and context-dependent effects. These shared barriers favor biomarker-guided patient selection and combination strategies rather than inhibition of a single lipid-metabolic target [202].

### 3.7. Metformin—An Antidiabetic Drug with Pleiotropic Antitumor Effects

Metformin exerts pleiotropic antitumor effects in EOC by disrupting mitochondrial bioenergetics, activating AMPK-dependent signaling, inhibiting mTOR, and restricting tumor-cell metabolic adaptability [203,204]. Inhibition of mitochondrial complex I reduces ATP production and increases the AMP/ATP ratio, leading to AMPK activation, suppression of mTOR-dependent growth and biosynthesis, cell-cycle arrest, and increased metabolic stress [204,205,206]. Metformin may also reduce H3K27 trimethylation by inhibiting the PRC2/EZH2 complex, although this effect appears more pronounced under normoglycemic conditions, suggesting that its activity depends on the metabolic context [207].

Metformin may additionally modulate antitumor immunity. It reduces the suppressive activity of myeloid-derived suppressor cells through AMPKα activation, HIF-1α suppression, and decreased CD39/CD73 expression [208]. It may also increase CD8+ T-cell infiltration and enhance granzyme B and IFN-γ production, providing a rationale for combination with PD-L1 blockade [209]. Other preclinical effects include suppression of pluripotency factors and Hedgehog signaling, sensitization of platinum-resistant cells to cisplatin [210], inhibition of TRIM37/TRAF2/NF-κB signaling [211], and reduced expression of MSLN, IL-6/STAT3, VEGF, and TGF-β1 [212].

Clinical evidence remains limited. In a nonrandomized phase II trial involving 38 patients with advanced EOC, metformin combined with chemotherapy was well tolerated, with median progression-free and overall survival of 18 and 57.9 months, respectively. Biological analyses showed a reduction in ALDH+CD133+ cancer stem cells and stromal changes associated with increased ex vivo cisplatin sensitivity, but the absence of randomization limits interpretation [213]. Meta-analyses of observational studies suggest an association between metformin use and reduced mortality, although prospective trials are required to establish therapeutic efficacy [214]. A population-based study of 4951 patients reported an association with survival among patients with diabetes, including those with serous carcinoma [215].

Combination strategies include metformin with CB-839 to target glucose and glutamine metabolism in KRAS-mutated ovarian cancer [181], with simvastatin to disrupt AMPK/mTOR signaling in ovarian cancer cells [216], and with PD-L1 inhibitors to enhance immune-cell infiltration and checkpoint blockade [209]. These findings support further evaluation of metformin as a metabolic and immunomodulatory adjunct [217].

The clinical translation of metformin is constrained by several shared barriers. Its antitumor activity appears to depend on systemic and tumor-specific metabolic conditions, while the effective exposure achieved in preclinical models may not be consistently reproduced in patients. Current clinical evidence is based largely on small nonrandomized trials and observational studies, which are vulnerable to patient selection and diabetes-related confounding. The absence of validated predictive biomarkers further limits the identification of patients most likely to benefit. Accordingly, metformin should remain an investigational adjunct rather than an established anticancer therapy in unselected EOC populations [218].

### 3.8. Targeting NAD+ Metabolism and Redox Homeostasis

EOC stem cells (OCSCs) maintain redox homeostasis through metabolic pathways that supply NADPH and glutathione, including glutamine, serine/glycine, and sulfur-containing amino acid metabolism. These pathways support antioxidant systems such as GPX4 and thioredoxin and protect tumor cells from oxidative stress and ferroptosis.

Therapeutic strategies under investigation include inhibition of the cystine transporter xCT to restrict glutathione synthesis, direct inhibition of GPX4 to promote lipid-peroxide accumulation, and targeting PHGDH, SHMT, or NAMPT to disrupt serine metabolism, NAD+ salvage, energy production, redox balance, and DNA repair [87,90,219].

However, clinical translation is complicated by the redundancy of antioxidant defense systems and the context-dependent effects of redox modulation. Tumor cells may compensate for inhibition of the xCT–GSH–GPX4 pathway by activating alternative ferroptosis defense mechanisms, while excessive disruption of redox homeostasis may also affect normal and immune cells. The dependence of ferroptosis sensitivity on tumor genotype, metabolic state, lipid composition, and microenvironment further complicates patient selection and supports the development of predictive biomarkers and rational combination strategies [220].

### 3.9. Epigenetic Therapies

#### 3.9.1. Rationale for Epigenetic Therapy in EOC

Epigenetic aberrations, including altered DNA methylation, histone modifications, and dysregulation of noncoding RNAs, are well-established contributors to the development and progression of EOC [96,100,221].

Of particular importance are epigenetic alterations that silence tumor suppressor genes through hypermethylation of CpG-rich promoter regions, as well as changes in the expression of genes involved in cell-cycle regulation, DNA repair, and apoptosis. Frequently affected genes include BRCA1, MLH1, RASSF1A, OPCML, ARHI/DIRAS3, PEG3, and PTEN [162,164,165]. Dysregulated expression of noncoding RNAs, particularly microRNAs, also contributes to EOC biology by altering mRNA stability and translation and thereby affecting tumor cell proliferation, metabolism, and invasive capacity [222,223].

Unlike DNA mutations, aberrant epigenetic modifications that suppress gene expression are potentially reversible, making them attractive therapeutic targets. Reactivation of epigenetically silenced tumor suppressor genes, such as *BRCA1*, *MLH1*, *RASSF1A*, *OPCML*, *ARHI* (DIRAS3), *PEG3*, and *PTEN,* may restore mechanisms controlling cell growth, DNA repair, and apoptosis [100,216].

#### 3.9.2. DNA Methyltransferase Inhibitors

Azacitidine (AZA) and decitabine (DAC) are cytosine nucleoside analogs that are incorporated into DNA during replication and form covalent complexes with DNA methyltransferases (DNMTs), thereby trapping and degrading the enzymes. Reduced DNMT activity leads to progressive demethylation of CpG regions during subsequent rounds of DNA replication, thereby enabling the re-expression of previously silenced genes [97,224]. This process may reactivate tumor suppressor genes and promote antitumor immune responses [98,99].

In EOC, DNMT inhibitors have attracted particular interest as agents that can resensitize tumor cells to platinum-based chemotherapy. In a phase I study by Fang et al., administration of decitabine before carboplatin reduced DNA methylation, including methylation within HOXA11 and BRCA1, and increased tumor cell sensitivity to treatment [224]. In a subsequent phase II study, the combination of decitabine and carboplatin demonstrated clinical activity in patients with recurrent EOC, although these findings require confirmation in larger randomized trials [225].

Not all clinical studies have confirmed the efficacy of this strategy. In a phase II trial by Glasspool et al., decitabine combined with carboplatin showed no meaningful clinical activity, and the study was terminated early because of hematologic toxicity and hypersensitivity reactions. The discrepant findings may have resulted, at least in part, from the use of a higher decitabine dose and a different dosing schedule [101].

Preclinical studies have also demonstrated synergistic activity between DNMT inhibitors and PARP inhibitors. Combining decitabine with PARP inhibition increased DNA damage, enhanced γH2AX formation, and impaired DNA repair, suggesting a potential strategy to sensitize EOC cells to PARP inhibitors. This approach is particularly relevant in the context of PARPi resistance because epigenetic modulation may alter the expression of genes involved in the DNA damage response [102].

#### 3.9.3. The “Viral Mimicry” Mechanism

DNA methyltransferase inhibitors (DNMTis) induce DNA demethylation, leading to the reactivation of endogenous retroviruses (ERVs) and other genomic repeat elements (REs), particularly long terminal repeat (LTR) sequences. Their transcription may generate double-stranded RNA (dsRNA), which is recognized by innate antiviral immune receptors, including the cytoplasmic RNA sensors MDA5 and RIG-I and the endosomal receptor TLR3. Activation of these receptors triggers MAVS-dependent signaling and the transcription factors IRF3, IRF7, and NF-κB, resulting in the production of type I interferons, including IFN-β, and the induction of antiviral response genes. This effect increases tumor cell immunogenicity and may sensitize EOC cells to immunotherapy, particularly immune checkpoint inhibitors [226,227].

The effect of DNMTis on RE expression depends on TP53 status. Cell lines harboring TP53 mutations show markedly fewer upregulated REs following epigenetic therapy than TP53-wild-type cell lines [226]. This observation has important clinical implications given the near-universal presence of TP53 mutations in HGSOC.

In murine EOC models, clinically relevant doses of azacitidine reduced the immunosuppressive tumor microenvironment through type I interferon signaling, increased the number of CD45+ cells and the proportions of activated CD8+ T cells and NK cells, and simultaneously decreased the proportions of macrophages and myeloid-derived suppressor cells [227].

Guadecitabine (SGI-110) is a decitabine prodrug with enhanced stability. In a phase II trial, guadecitabine combined with pembrolizumab was evaluated in 35 patients with platinum-resistant EOC, yielding a partial response rate of 8.6% and stable disease in 22.9% of patients, with a median duration of clinical benefit of 6.8 months. Correlative analyses demonstrated LINE-1 hypomethylation in peripheral blood mononuclear cells after treatment, activation of antitumor immunity in post-treatment biopsies, and an association between durable clinical benefit and higher frequencies of naïve and central memory CD4+ T cells and classical monocytes [228].

#### 3.9.4. Histone Deacetylase Inhibitors

Histone deacetylase inhibitors (HDACis) are among the best-characterized epigenetic therapeutic strategies. By inhibiting the removal of acetyl groups from histones, they increase chromatin acetylation, promote chromatin relaxation, and reactivate previously silenced genes, including those involved in cell-cycle control and antitumor responses. In EOC cells, HDACis exert pleiotropic antiproliferative, proapoptotic, and anti-invasive effects. They induce cell-cycle arrest by increasing the expression of the cyclin-dependent kinase inhibitor p21^WAF1 and modulating regulators of cell-cycle phase transitions, such as cyclin B1 and p34^CDC2, thereby leading to accumulation in the G0/G1 or G2/M phases. In addition, HDACis induce apoptosis by activating caspases-3, -8, and -9; enhancing p53-dependent signaling; and increasing PARP cleavage. They also exert anti-invasive effects by reducing the expression of extracellular matrix metalloproteinases, including MMP-9, and inhibiting cytoskeletal changes associated with cell migration [229,230,231,232].

SAHA (vorinostat) is a pan-HDAC inhibitor that inhibits proliferation, induces S/G2-phase arrest in SKOV3 and SKOV3/DDP cells or G1-phase arrest in HO8910 and HO8910-PM cells, promotes apoptosis, and reduces the migration and invasion of EOC cells, including cisplatin-resistant cells, in preclinical studies [229]. The combination of SAHA and decitabine synergistically inhibited EOC cell growth by inducing apoptosis, G2/M cell-cycle arrest, and autophagy, and by reactivating the tumor suppressor genes ARHI and PEG3 [233].

Panobinostat, a potent pan-HDAC inhibitor, suppressed EOC cell growth, induced cell-cycle arrest and apoptosis, increased the levels of cleaved PARP and caspase-3, promoted H2B acetylation, and increased γH2AX expression in preclinical studies. In an orthotopic EOC model, panobinostat was as effective as carboplatin in prolonging survival, while the combination of both agents produced synergistic antitumor effects [234].

Valproic acid (VPA), an inhibitor of class I and IIa HDACs, has demonstrated antiproliferative and proapoptotic activity in ovarian cancer cell models [232,235].

Despite favorable preclinical findings, the clinical translation of HDAC inhibitors in EOC remains challenging. In a phase II trial, sodium valproate combined with etoposide in patients with platinum-resistant EOC did not improve the response rate compared with historical outcomes for etoposide monotherapy, and the study was terminated early. However, the trial was limited by its small sample size and nonrandomized design [236]. In another phase II trial, belinostat combined with carboplatin showed no meaningful clinical activity in patients with platinum-resistant EOC, indicating the need for further optimization of dosing regimens and identification of predictive biomarkers of treatment response [237]. Similarly, the combination of vorinostat, carboplatin, and gemcitabine was limited by hematologic toxicity, preventing determination of an optimal therapeutic dose [238].

ARID1A mutations, which occur in more than 50% of clear cell EOCs, confer sensitivity to pan-HDAC inhibitors such as SAHA. Mechanistically, HDAC2 cooperates with EZH2 in an ARID1A-dependent manner and functions as an EZH2 corepressor in the silencing of tumor suppressor genes, including PIK3IP1. In orthotopic and genetically engineered models of ARID1A-inactivated clear cell EOC, SAHA reduced tumor growth and ascites formation and significantly prolonged survival [239].

#### 3.9.5. EZH2 Inhibitors

EZH2 is the catalytic subunit of the polycomb repressive complex 2 (PRC2), responsible for histone H3 lysine 27 trimethylation (H3K27me3) and the silencing of tumor suppressor genes. EZH2 is frequently overexpressed in EOC and is associated with advanced-stage disease, metastasis, and poorer prognosis [240,241,242].

Tazemetostat is the first EZH2 inhibitor approved by the FDA for the treatment of epithelioid sarcoma and follicular lymphoma and is currently being investigated in EOC [243]. Novel covalent EZH2 inhibitors based on the tazemetostat scaffold, including SKLB-03220, have demonstrated potent and selective EZH2 inhibition, marked suppression of EOC cell-line growth, and sustained depletion of H3K27me3 after drug washout. In a PA-1 xenograft model, oral administration of SKLB-03220 significantly inhibited tumor growth without apparent adverse effects [244].

EZH2 inhibition sensitizes homologous recombination-proficient EOC to PARP inhibitors in a CARM1-dependent manner. CARM1 promotes MAD2L2 silencing by inducing a switch from SWI/SNF-mediated regulation to EZH2-dependent repression through methylation of the BAF155 subunit. EZH2 inhibition increases MAD2L2 expression and reduces DNA end resection, thereby enhancing nonhomologous end joining, increasing chromosomal abnormalities, and ultimately inducing mitotic catastrophe in HR-proficient cells treated with PARP inhibitors. EZH2 inhibition sensitized EOC with high, but not low, CARM1 expression to PARP inhibitors in both orthotopic and patient-derived xenograft models [245]. Regarding the dual inhibition of HOTAIR and EZH2, HOTAIR, a long noncoding RNA overexpressed in HGSOC, recruits EZH2 to promote H3K27 trimethylation and the silencing of tumor suppressor genes. HOTAIR knockout resensitized EOC cells to platinum agents and reduced the cancer stem cell population. Combining HOTAIR inhibition with an EZH2 inhibitor and platinum-based chemotherapy reduced tumor formation and prolonged survival in vivo [246].

#### 3.9.6. BET Inhibitors

BET proteins, including BRD2, BRD3, and BRD4, recognize acetylated histones and regulate gene transcription, including that of the c-MYC and FOXM1 oncogenes. BET inhibitors, including JQ1 and other bromodomain inhibitors, prevent recognition of acetylated histones, leading to the suppression of oncogenes and induction of apoptosis [247,248,249].

In preclinical studies, the BET inhibitor JQ1 demonstrated broad antitumor effects across EOC subtypes. Unlike many other malignancies in which sensitivity to BET inhibition results from suppression of super-enhancer-dependent MYC expression, JQ1-sensitive EOC cells showed marked disruption of the FOXM1 pathway, a major driver of EOC progression. JQ1 demonstrated in vivo efficacy in both cell line-derived xenograft and patient-derived xenograft models [249].

c-MYC amplification as a target for BET inhibition. Analysis of the mutational landscape of 64 primary, 41 metastatic, and 17 recurrent EOC tumors showed that c-MYC amplifications were acquired in recurrent disease. Primary cell lines and xenografts derived from chemoresistant tumors were sensitive to JQ1 and GS-626510, suggesting that BET inhibition may represent a potential therapeutic strategy for patients with recurrent or chemoresistant EOC [247].

The combination of the BET inhibitor HJP-178 and the METTL3 inhibitor STM2457 synergistically suppressed proliferation across several EOC cell lines. Mechanistically, the combination reduced SP1 RNA and protein levels via both transcriptional and post-transcriptional mechanisms, leading to decreased BCL-2 expression and activation of caspase-dependent apoptosis [250]. Although most evidence concerning BET inhibitors in EOC currently derives from preclinical studies, these approaches remain promising therapeutic strategies requiring further clinical evaluation. Combinations of BET inhibitors with other targeted agents represent a potential direction for future investigation.

#### 3.9.7. Combination Therapy: Epigenetic Modulation and Immunotherapy

Combined epigenetic therapy involving DNMT and HDAC inhibition enhanced antitumor immune responses and reduced tumor burden in preclinical EOC models [227,251]. Epigenetic therapy may convert immunologically “cold” tumors into “hot” tumors by inducing interferon signaling and increasing lymphocyte infiltration, thereby sensitizing tumors to immune checkpoint blockade [252,253,254].

In a phase II trial involving 35 patients with platinum-resistant EOC, guadecitabine at 30 mg/m^2^ on days 1–4, combined with pembrolizumab at 200 mg intravenously on day 5 of each 21-day cycle, achieved a partial response rate of 8.6% and stable disease in 22.9% of patients. The median duration of clinical benefit was 6.8 months. Correlative analyses demonstrated LINE-1 hypomethylation in peripheral blood mononuclear cells after treatment, activation of antitumor immunity in post-treatment biopsies, and an association between durable clinical benefit and higher frequencies of naïve and central memory CD4+ T cells and classical monocytes [228].

The combination of a DNMT inhibitor and an HDAC6 inhibitor enhanced type I interferon responses, increased cytokine and chemokine expression, and upregulated components of the MHC class I antigen-presentation machinery in EOC cell lines. In the ID8 Trp53^−/−^ murine model, combined DNMT and HDAC6 inhibition increased the abundance of tumor-killing immune cells, including IFN-γ+ CD8+ T cells, NK cells, and NKT cells, while reversing the immunosuppressive tumor microenvironment by reducing myeloid-derived suppressor cells and PD-1^high CD4+ T cells. These immune changes were associated with prolonged survival [251].

### 3.10. Genome Editing and Gene Therapy

#### 3.10.1. CRISPR/Cas9 Genome Editing

The CRISPR/Cas9 system enables precise editing of genes involved in EOC progression and drug resistance. Investigated strategies include correction of tumor suppressor alterations, such as BRCA1/2 or TP53 mutations, and targeting genes involved in ovarian cancer progression and therapy resistance. However, most of these approaches remain experimental and at the preclinical stage [255,256,257]. Epigenetic regulators also represent potential therapeutic targets. In ovarian cancer models, CRISPR/Cas9-mediated targeting of DNMT1 induced DNA demethylation and reactivated silenced tumor suppressor genes [258].

Efficient delivery of CRISPR/Cas9 components to tumor cells remains critical to the success of these therapies. Delivery platforms under development include lipid nanoparticles, polymeric nanoparticles, functionalized gold nanoparticles, extracellular vesicles, and viral vectors, including adeno-associated viruses and lentiviruses [255,256,257]. Despite the variety of available platforms, effective tumor-directed delivery therefore remains a shared translational challenge for CRISPR/Cas9-based approaches in EOC.

Folate-functionalized liposomes carrying a CRISPR plasmid coexpressing Cas9 and a DNMT1-targeting single-guide RNA reduced DNMT1 expression and inhibited tumor growth in paclitaxel-sensitive and paclitaxel-resistant EOC models, while producing fewer adverse effects than paclitaxel [258]. PtUTP-F nanoparticles delivering a CRISPR/dCas9 activation system targeting CT45 inhibited PP4C activity, disrupted DNA repair pathways, and increased sensitivity to platinum-based agents [259]. These studies demonstrate the potential of targeted delivery systems, but both approaches remain preclinical.

#### 3.10.2. Adeno-Associated Virus (AAV)-Based Gene Therapy

Adeno-associated virus (AAV) vectors represent a potential platform for delivering therapeutic genes to EOC cells. Advances in AAV capsid engineering are intended to improve delivery selectivity, including targeting EOC stem cells identified by specific surface markers [260]. AAV-based gene therapy includes the delivery of genes encoding antiangiogenic factors, such as endostatin, angiostatin, and thrombospondin; immune-activating factors, including IL-2, IL-12, GM-CSF, and IFN-γ; and functional copies of tumor suppressor genes, such as TP53 and PTEN. These approaches aim to inhibit neovascularization, modulate the tumor microenvironment, or restore mechanisms regulating tumor-cell proliferation and survival [261,262].

However, the clinical translation of AAV-based cancer gene therapy remains limited by insufficient tumor-selective transduction, host immune responses to the vector or its transgene product, packaging capacity constraints, and challenges in scalable vector production [261].

### 3.11. Physical and Locoregional Treatment Modalities

#### 3.11.1. Hyperthermic Intraperitoneal Chemotherapy

Hyperthermic intraperitoneal chemotherapy (HIPEC) involves a single intraoperative administration of a heated chemotherapeutic solution directly into the peritoneal cavity following cytoreductive surgery [106,107].

The mechanisms of action of HIPEC involve a multifactorial therapeutic effect resulting from both hyperthermia and the high local concentration of chemotherapy within the peritoneal cavity. A key component is the direct cytotoxic effect of maintaining the temperature at 41 ± 1 °C, which damages cellular structures and induces proteotoxic stress in tumor cells. Hyperthermia enhances the cytotoxic effects of chemotherapy by increasing drug penetration into superficial tumor deposits, altering cellular stress responses, and promoting tumor cell death, particularly in peritoneal dissemination. HIPEC may also increase tumor susceptibility to platinum-based chemotherapy through enhanced cellular stress and apoptotic signaling. In addition, HIPEC modulates the tumor microenvironment by reducing the number of activated fibroblasts associated with epithelial–mesenchymal transition and MMP-11-expressing cancer-associated fibroblasts, thereby potentially limiting tumor invasiveness [263].

Because of the complexity of HIPEC and the need for extensive cytoreductive surgery, appropriate patient selection is critical to treatment efficacy and safety. HIPEC may be considered in patients with EOC involving the peritoneum when optimal or complete cytoreduction can be achieved. Factors limiting eligibility include advanced age, particularly >70 years; severe comorbidities, especially cardiovascular, respiratory, neurologic, or renal disease; deterioration in general condition during systemic chemotherapy; malnutrition; concomitant extra-abdominal metastases; unresectable liver metastases; extensive retroperitoneal disease with lymph-node involvement; and contraindications to chemotherapy [264].

Appropriate preoperative assessment may reduce the incidence of severe complications and perioperative mortality, which is particularly important because the procedure combines extensive surgery with the locoregional administration of cytotoxic agents under hyperthermic conditions [265].

Accurate assessment of EOC stage and the extent of peritoneal involvement is essential when determining eligibility for cytoreductive surgery combined with HIPEC. Direct surgical inspection during laparoscopy or laparotomy remains the most reliable method for evaluating peritoneal lesions. FDG-PET/CT may help identify distant metastases in patients being considered for HIPEC, although its sensitivity for small, millimeter-sized peritoneal deposits remains limited. The Peritoneal Cancer Index (PCI) is an important prognostic and eligibility-assessment tool that quantifies the extent and distribution of tumor lesions across 13 anatomic regions of the abdominal cavity. Accurate preoperative estimation of the PCI is relevant to predicting whether complete cytoreduction can be achieved. Studies indicate that magnetic resonance imaging may be more accurate than computed tomography in predicting PCI values and evaluating peritoneal dissemination, potentially improving patient selection for surgery combined with HIPEC [265]. The development of intraoperative imaging techniques may further improve the precision of cytoreductive procedures. Near-infrared imaging enables intraoperative mapping of sentinel lymph nodes, identification of tumor lesions, and visualization of anatomically important structures. Of particular interest in EOC is the use of fluorophores targeting folate receptor alpha, which may facilitate the intraoperative visualization of peritoneal metastases [266].

#### 3.11.2. Rationale and Clinical Evidence for HIPEC

One of the principal advantages of intraperitoneal chemotherapy is the ability to achieve substantially greater tumor exposure to chemotherapeutic agents than with intravenous administration. The rationale for performing cytoreductive surgery before chemotherapy is the substantial biological heterogeneity of advanced EOC, including hypoxic regions and treatment-resistant cell populations. Maximal cytoreduction decreases tumor burden, thereby improving the effectiveness of systemic treatment and increasing the likelihood of achieving a complete response to chemotherapy [267,268].

Direct administration of drugs into the peritoneal cavity produces markedly higher local concentrations while limiting systemic exposure. For agents used in EOC, the ratio of intraperitoneal to plasma drug concentrations may reach approximately 18–20 for cisplatin and carboplatin and more than 100–1000 for taxanes, including paclitaxel and docetaxel [269]. This substantial pharmacokinetic advantage underlies the greater efficacy of locoregional treatment in disease predominantly confined to the peritoneal cavity [270].

Earlier randomized trials, including GOG-104, GOG-114, and GOG-172, evaluated conventional intraperitoneal chemotherapy rather than HIPEC. These studies established the pharmacologic and clinical rationale for intraperitoneal drug delivery in optimally cytoreduced ovarian cancer and demonstrated that locoregional administration could improve treatment outcomes compared with exclusively intravenous therapy. In GOG-172, for example, patients receiving an intraperitoneal cisplatin- and paclitaxel-containing regimen had longer overall survival than those treated with intravenous chemotherapy. However, these trials did not incorporate hyperthermia or intraoperative perfusion and therefore should not be interpreted as direct evidence of HIPEC efficacy [271]. Direct clinical evidence for HIPEC should therefore be considered separately and derives from randomized trials specifically evaluating intraoperative hyperthermic intraperitoneal chemotherapy, including OVHIPEC-1 and CHIPOR.

Despite greater locoregional toxicity, including abdominal pain, peritonitis, catheter-related infections, adhesions, nausea, vomiting, and drug-dependent nephrotoxicity and neurotoxicity, quality of life at 12 months was comparable between patients receiving intraperitoneal and intravenous treatment [272]. Long-term follow-up suggests that the benefits of intraperitoneal chemotherapy may persist for many years and that completing more planned treatment cycles is associated with improved overall survival. Cisplatin, carboplatin, and paclitaxel are the agents most commonly used for intraperitoneal treatment of EOC [273,274].

#### 3.11.3. Clinical Evidence and Guideline Recommendations for HIPEC

OVHIPEC-1 was a randomized phase III trial evaluating the addition of HIPEC (cisplatin at 100 mg/m^2^ at 41 ± 1 °C for 90 min) to interval debulking surgery in patients with stage III EOC following neoadjuvant chemotherapy. In the final survival analysis, after a median follow-up of 10.1 years, HIPEC provided a significant clinical benefit. Median recurrence-free survival was 14.3 months in the HIPEC group compared with 10.7 months in the control group (HR, 0.63; *p* < 0.001). Median overall survival was also longer in the intervention group (44.9 months) than in the control group (33.3 months; HR, 0.70; *p* = 0.011). No meaningful imbalance was observed between the study groups in the use of subsequent lines of therapy after recurrence, supporting the robustness of the survival findings [106].

CHIPOR was a randomized phase III trial evaluating HIPEC during secondary cytoreductive surgery in patients with a first late recurrence of EOC, defined as recurrence occurring at least 6 months after the last platinum-based chemotherapy. After a median follow-up of 6.2 years, the study demonstrated a survival benefit. In CHIPOR, HIPEC consisted of intraperitoneal cisplatin administered at 41 ± 1 °C during secondary cytoreductive surgery. HIPEC significantly improved overall survival compared with cytoreductive surgery alone, with a median OS of 54.3 versus 45.8 months (HR, 0.73; *p* = 0.024) [103].

A meta-analysis of eight randomized controlled trials involving 1259 patients showed that HIPEC significantly improved overall survival (HR, 0.76; *p* = 0.009). A significant benefit was observed in the subgroup with primary EOC (HR, 0.66; *p* = 0.001), but not in the subgroup with recurrent EOC (HR, 0.87; *p* = 0.38) [104]. An updated meta-analysis presented as a conference abstract included 12 studies involving 1893 patients and reported an HR of 0.67 for overall survival (*p* < 0.00001), with similar benefits in previously untreated patients (HR, 0.67) and those undergoing secondary cytoreductive surgery (HR, 0.66) [108]. The most recent meta-analysis of seven randomized controlled trials involving 1300 patients, based on reconstructed time-to-event data, demonstrated a significant benefit of HIPEC in newly diagnosed EOC patients treated with interval debulking surgery, for both progression-free survival (HR, 0.65) and overall survival (HR, 0.68). However, no significant benefit was observed in recurrent EOC [105].

According to the NCCN Guidelines, version 3.2026, HIPEC with cisplatin at 100 mg/m^2^ may be considered during interval debulking surgery for stage III EOC and for appropriately selected patients with stage IV disease who have shown a favorable response to neoadjuvant chemotherapy; this recommendation is classified as category 2B. Sodium thiosulfate may be administered at the beginning of perfusion and subsequently as a continuous infusion to reduce renal toxicity during HIPEC [59].

#### 3.11.4. Pressurized Intraperitoneal Aerosol Chemotherapy

Pressurized intraperitoneal aerosol chemotherapy (PIPAC) is an innovative, minimally invasive method of delivering intraperitoneal chemotherapy as a pressurized aerosol during laparoscopy. Compared with HIPEC, PIPAC offers several potential advantages arising from its distinct method of drug delivery into the peritoneal cavity. These include improved intraperitoneal drug distribution, resulting in more uniform contact between the cytotoxic agent and tumor surfaces, and deeper penetration into tumor tissue than that achieved with conventional intraperitoneal techniques [109].

PIPAC also uses substantially lower doses of chemotherapeutic agents, such as cisplatin at 10.5 mg/m^2^ and doxorubicin at 2.1 mg/m^2^, thereby limiting systemic toxicity. The procedure may be repeated at 4–6-week intervals, allowing multiple therapeutic exposures over the course of treatment. Another important advantage is the ability to objectively assess histologic response using the Peritoneal Regression Grading Score (PRGS), which enables monitoring of treatment efficacy across successive cycles [109].

#### 3.11.5. Clinical Evidence for PIPAC in EOC

A US phase I trial (n = 15) reported that PIPAC with cisplatin and doxorubicin achieved an 86.7% completion rate across at least two treatment cycles. Laparoscopic and histologic responses were observed in 30.8% and 46.2% of patients, respectively, and median OS was 17.1 months [110].

An Italian multicenter study (n = 40) showed that PIPAC was feasible in patients with platinum-resistant or recurrent EOC and peritoneal dissemination, with an acceptable safety profile and the potential for repeated administration. Signals of antitumor activity were observed, including improved ascites control in some patients after the initial treatment cycles and objective responses assessed using available clinical and histopathologic criteria. These findings provide an early signal of clinical activity in highly advanced, platinum-resistant EOC; however, interpretation is limited by the small, nonrandomized study population, and confirmation in adequately powered prospective trials is required [111]. The PARROT study (n = 43) revealed that in patients with platinum-resistant recurrent EOC, PIPAC achieved a clinical benefit rate of 82%, a median OS of 27 months, and preserved quality of life [112]. A randomized trial from India revealed, in turn, that compared with intravenous chemotherapy in patients with platinum-resistant EOC, PIPAC produced a significantly higher objective response rate (66.6% vs. 22.5%), a lower incidence of grade ≥ 3 adverse events (10.0% vs. 35.7%), and better quality of life [113].

Real-world data from the international ISSPP PIPAC database further support the feasibility and safety of PIPAC-directed therapy in patients with peritoneal metastases. In the third annual report, 1126 patients underwent 3224 PIPAC procedures across 17 centers. The median Peritoneal Cancer Index remained stable during repeated treatments, whereas histopathologic response assessed by the Peritoneal Regression Grading Score improved over successive PIPAC cycles, with complete or major responses (mean PRGS ≤ 2) observed in 57%, 72%, and 75% of patients at PIPAC 1, 2, and 3, respectively. Major procedure-related complications were uncommon [275].

#### 3.11.6. Exploratory Locoregional and Physical Modalities

Thermal ablation techniques, including radiofrequency ablation (RFA), microwave ablation (MWA), and cryoablation, have been evaluated mainly in small retrospective series involving selected patients with metastatic gynecologic malignancies or EOC liver metastases. In a study of 42 patients with 119 tumors, including 27 patients with EOC, the overall technical success rate was 96.2%, complete response after the first ablation was achieved in 95.6% of patients, and major adverse events occurred in 4.8% [276]. In a retrospective study of 60 patients with EOC liver metastases, RFA combined with chemotherapy was associated with higher 1-, 2-, and 3-year survival rates than chemotherapy alone [277]. MWA produced oncologic outcomes comparable to those of surgical resection in a small series, while reducing operative time, blood loss, hospital length of stay, and treatment costs [278]. Other small studies also reported high technical success and favorable local control after RFA for hepatic or retroperitoneal metastatic disease [279,280]. However, these findings are limited by small sample sizes, retrospective design, heterogeneous populations, and substantial selection bias.

Cryoablation may additionally exert immunomodulatory effects by inducing tumor-cell necrosis and releasing tumor antigens and damage-associated molecular patterns, including HMGB1, ATP, calreticulin, and HSP70. These events may promote dendritic cell maturation, effector T-cell activation, and macrophage polarization toward a proinflammatory phenotype, providing a biological rationale for combining cryoablation with immune checkpoint inhibition and for inducing an abscopal response [281,282,283,284]. In a patient with clear cell EOC and pulmonary metastases, cryoablation of a single lesion, followed by pembrolizumab, was associated with complete regression of the remaining pulmonary metastases [282]. Although this observation suggests a potential synergistic effect, it remains an isolated case report. Similarly, an exploratory study of nine patients with advanced solid tumors resistant to immune checkpoint inhibitors reported an objective response rate of 22.2% after cryoablation combined with intra-arterial anti-PD-1 perfusion [285]. These findings are hypothesis-generating and do not establish clinical efficacy in EOC. Experimental data also support the investigation of additional immune checkpoints, including VISTA, although their role in combination with cryoablation remains uncertain [281].

Modulated electro-hyperthermia (mEHT), also known as oncothermia, is designed to preferentially deposit energy within tumor tissue by exploiting differences in the thermal and bioelectromagnetic properties of malignant and normal cells [286,287,288]. Preclinical studies suggest that mEHT can induce caspase-dependent apoptosis, PARP cleavage, p38 MAPK activation, calreticulin exposure, and HSP70 release, thereby promoting immunogenic cell death [286,287,289,290,291]. mEHT may also reduce protumorigenic CAF populations and enhance the effects of cisplatin or autophagy inhibition in experimental EOC models [263,289,292]. In vitro, mEHT induced greater apoptosis than conventional hyperthermia at comparable temperatures, although these findings were partly derived from non-EOC models [291].

Clinical evidence for mEHT in EOC remains limited. In a phase I/II study involving 19 patients with recurrent or progressive disease, mEHT was feasible and caused no procedure-related complications, but stable disease was achieved in only 12.5% of patients, with median progression-free and overall survival of 4.0 and 8.0 months, respectively [293]. A retrospective cohort of 22 patients treated with chemotherapy and mEHT reported high response rates and prolonged remission in a proportion of patients [294]. However, these results are difficult to interpret due to the small sample size, retrospective design, heterogeneous population, absence of a control group, and the potential contribution of concomitant chemotherapy. Accordingly, the clinical value of mEHT in EOC remains uncertain.

Photodynamic therapy (PDT) and sonodynamic therapy (SDT) generate cytotoxic reactive oxygen species following activation of a photosensitizer by light or a sonosensitizer by ultrasound, respectively. PDT may induce mitochondrial damage, lipid peroxidation, vascular injury, apoptosis, and immunogenic cell death [295,296,297]. Mitochondria-targeted PDT and photoimmunoconjugates directed against EOC-associated antigens have been proposed as strategies for treating residual peritoneal disease while reducing off-target toxicity [295,297]. The photosensitizer TMPyP4 exhibited enhanced activity in the presence of hydrogen peroxide, leading to increased ROS generation, mitochondrial dysfunction, DNA damage, and HIF-1α degradation in EOC cells [298].

SDT offers greater tissue penetration than PDT and may be more suitable for deeply located lesions. Its effects are mediated by acoustic cavitation, sonoluminescence, and ROS production, which can directly damage tumor cells and increase vascular permeability [299,300,301,302]. Nanoparticle-based systems have been developed to improve oxygen delivery, tumor targeting, and immune activation. IRO@FA nanoparticles targeting folate receptors induced apoptosis and immunogenic cell death in ID8 models and increased dendritic-cell maturation and T-cell infiltration [303]. OIX nanoparticles containing oxygen, indocyanine green, and oxaliplatin inhibited both primary and distant tumors in a bilateral murine EOC model and increased cytotoxic T-cell infiltration [304]. Nevertheless, the evidence for both PDT and SDT in EOC remains predominantly preclinical, and clinical translation is limited by tumor hypoxia, delivery challenges, treatment standardization, and the lack of comparative clinical studies.

Irreversible electroporation (IRE) uses short, high-intensity electrical pulses to disrupt cell membranes permanently and induce predominantly nonthermal cell death, while largely preserving the extracellular matrix, vascular structures, and neural tissue [305]. High-frequency irreversible electroporation (H-FIRE) uses short bipolar pulses and may reduce muscle contractions and Joule heating while allowing more precise control of the ablation zone [306,307]. In EOC, however, both approaches remain at the preclinical stage. Experimental studies using the mouse ovarian surface epithelial model suggest that optimized H-FIRE parameters may preferentially eliminate malignant cells and tumor-initiating cell populations while sparing normal epithelial cells [308,309,310]. Elimination of these populations may be relevant because of their capacity for self-renewal, tumor reconstitution, drug efflux, enhanced DNA repair, and entry into quiescence [310,311].

IRE may also induce immunogenic cell death, release tumor antigens and DAMPs, activate dendritic cells, and enhance T-cell responses, providing a rationale for combination with immunotherapy [312,313,314]. However, the reproducibility, selectivity, safety, and clinical relevance of these effects in EOC remain unproven. Further development will require optimizing pulse parameters and conducting a prospective evaluation of clinical feasibility and efficacy.

Collectively, these exploratory locoregional and physical modalities are supported by substantially weaker evidence than HIPEC and should not be regarded as clinically equivalent. Thermal ablation has shown feasibility in carefully selected patients with localized metastatic disease, whereas cryoablation combined with immunotherapy, mEHT, PDT, SDT, and IRE/H-FIRE remain supported mainly by small uncontrolled studies, isolated case reports, or preclinical models. Their future evaluation should prioritize standardized treatment protocols, clinically relevant patient selection, prospective comparative studies, and outcomes that distinguish local tumor control from meaningful effects on systemic resistance and survival.

### 3.12. Targeted Drug-Delivery Systems in EOC

Nanomedicine enables the development of drug-delivery systems intended to improve the bioavailability, stability, controlled release, and tumor selectivity of anticancer agents while reducing systemic toxicity. The principal platforms investigated in EOC include liposomes, dendrimers, polymeric nanoparticles, polymeric micelles, and lipid nanoparticles. Their size, morphology, surface composition, and ligand functionalization can be modified to improve interactions with tumor cells, including through active targeting of receptors overexpressed in EOC [315,316].

Nanocarriers may address the poor aqueous solubility, rapid elimination, limited tumor penetration, and systemic toxicity of conventional anticancer agents by improving drug stability, prolonging circulation, and enhancing tumor accumulation. Passive accumulation may occur through the enhanced permeability and retention effect, whereas active targeting uses ligands, antibodies, or peptides that recognize tumor-associated receptors [317]. Liposomes are among the best-characterized platforms and can encapsulate hydrophilic compounds within their aqueous core and lipophilic compounds within the lipid bilayer [318]. PEGylation increases their stability and systemic exposure by limiting rapid uptake by the reticuloendothelial system [319].

PEGylated liposomal doxorubicin represents a clinically established application of nanomedicine in EOC. Liposomal encapsulation prolongs doxorubicin circulation, reduces clearance, promotes tumor accumulation, and limits systemic toxicity [320]. PEGylated paclitaxel-containing liposomes have also inhibited EOC-cell proliferation and induced apoptosis in preclinical studies [321].

More complex platforms include hybrid exosome–liposome systems and solid lipid nanoparticles. A hybrid system co-delivering microRNA-497 and triptolide was developed to increase therapeutic efficacy and overcome chemoresistance in EOC [322]. In preclinical models, this platform improved cargo delivery, inhibited tumor growth, and modulated the tumor microenvironment, which included increasing M1-like and reducing M2-like macrophages [322]. Solid lipid nanoparticles provide controlled release and protection of encapsulated agents from degradation [323]. Paclitaxel-loaded solid lipid nanoparticles prolonged drug exposure within the peritoneal cavity and showed greater preclinical antitumor activity than conventional formulations [324].

Nanocarriers have also been investigated as tools for targeting metabolic dependencies. Folate-functionalized FA-DCNP nanoparticles co-encapsulating the glutamine-metabolism inhibitor DON and calcium carbonate released DON and Ca^2+^ in the acidic tumor microenvironment. This approach simultaneously inhibited glutamine metabolism, induced calcium overload, and promoted M1 macrophage polarization in preclinical EOC models [325].

Despite extensive preclinical development, the translation of nanocarrier systems remains limited by shared biological barriers. Nanoparticle accumulation through the EPR effect varies among tumor types, lesions, and individual patients, while systemic clearance by the liver and spleen can substantially reduce the dose reaching the tumor. Nanocarriers must also penetrate the tumor interstitium, be taken up by cells, evade intracellular degradation, and release their cargo at the intended site of action. Differences between commonly used animal models and human tumors further limit the predictive value of preclinical findings [326].

Overall, most EOC-directed nanocarriers, including lipid, polymeric, micellar, hydrogel, and hybrid platforms, remain at the in vitro or in vivo preclinical stage. Although multidrug co-delivery and simultaneous targeting of tumor cells, resistance pathways, and the tumor microenvironment have shown preclinical potential, their clinical value remains unproven and will depend on overcoming the shared delivery and translational barriers described above [327].

## 4. Future Directions

**Figure 1:**
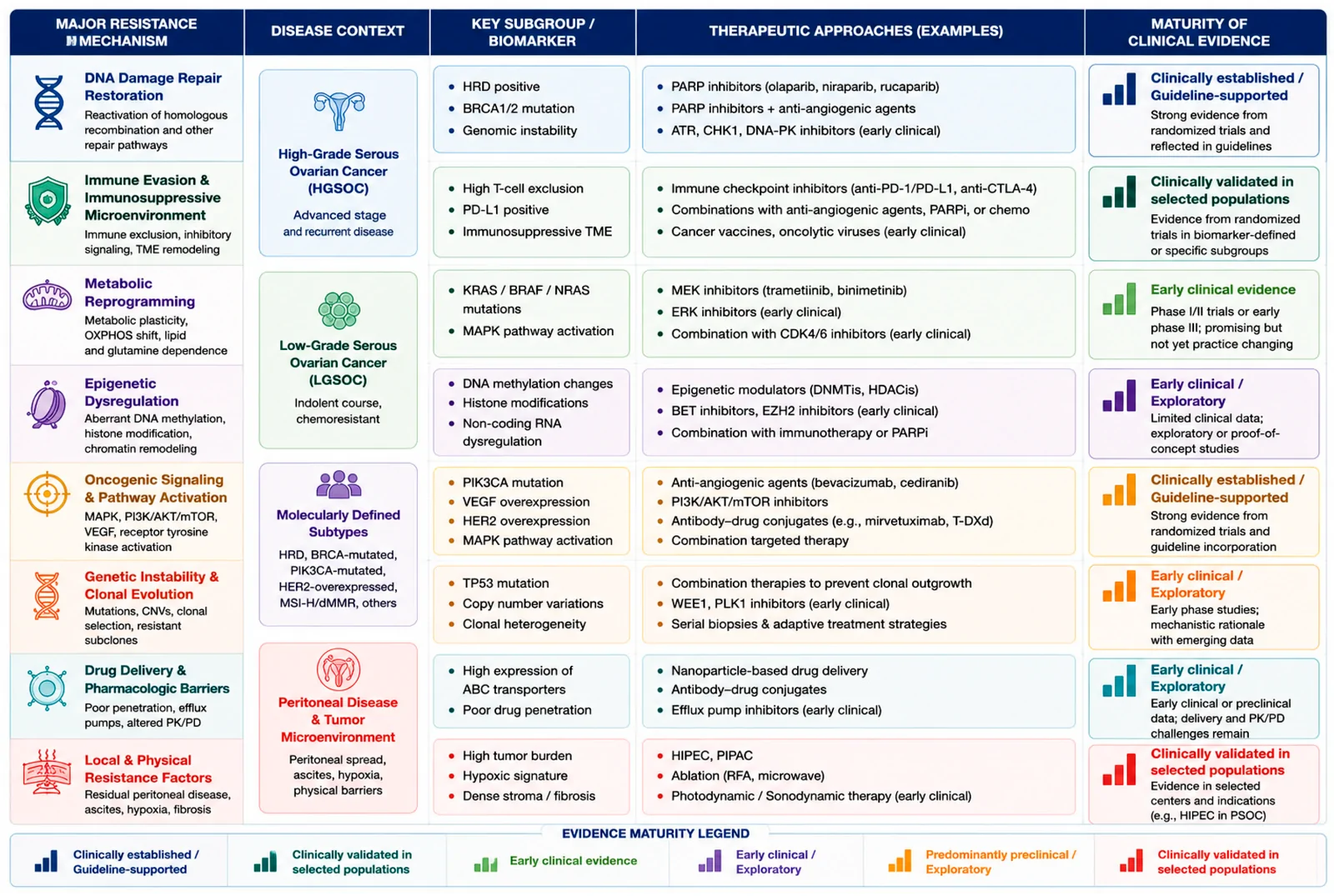
Integrated framework linking mechanisms of therapy resistance in epithelial ovarian cancer with disease context, biomarker-defined subgroups, therapeutic strategies, and maturity of clinical evidence.

The growing understanding of the molecular biology of EOC has enabled therapeutic strategies to be aligned more closely with specific mechanisms of treatment resistance, disease context, histologic or biomarker-defined subgroups, and the maturity of the available clinical evidence (Figure 1). These strategies include molecularly targeted therapies, immunotherapy, metabolic and epigenetic interventions, cellular and gene-based approaches, drug-delivery platforms, and locoregional or physical modalities. Their clinical relevance varies substantially, ranging from guideline-supported or clinically validated treatments in selected populations to early clinical and predominantly preclinical or exploratory approaches.

Future research should address several questions arising directly from the available evidence. In PARP inhibitor therapy, it remains necessary to determine how resistance mechanisms, including restoration of homologous recombination, replication-fork stabilization, drug efflux, and alterations in alternative DNA-repair pathways, can be detected and therapeutically overcome. For antiangiogenic therapy, a major unresolved question is whether any of the investigated biomarkers, including VEGF-related factors, the Tie2/Ang1 axis, inflammatory mediators, or molecular EOC subtypes, can reliably identify patients most likely to benefit. Similarly, immunotherapy requires more effective biomarkers than PD-L1 expression alone, given the low response rates to immune-checkpoint inhibitor monotherapy and the inconsistent results of combination trials.

For antibody–drug conjugates and adoptive cell therapies, a key testable question is how heterogeneous antigen expression, limited tumor penetration, the immunosuppressive peritoneal microenvironment, and treatment-related toxicity influence treatment efficacy and patient selection. In metabolic therapy, the key challenge is to identify tumors that are sufficiently dependent on glycolysis, oxidative phosphorylation, glutamine, arginine, or lipid metabolism to permit biomarker-guided treatment. Epigenetic therapies require clarification of why encouraging preclinical findings have not consistently translated into clinical benefit and which molecular contexts may predict response to DNMT, HDAC, EZH2, or BET inhibition.

Finally, the clinical role of early-stage strategies, including CAR-T and CAR-NK therapies, CRISPR/Cas9-based approaches, nanocarriers, PIPAC, and experimental physical modalities, must be defined through studies that establish safety, reproducibility, appropriate patient selection, and comparative efficacy. Future progress will therefore depend not only on the development of new treatments, but also on identifying the molecular and clinical settings in which each strategy is most likely to be effective.

## 5. Conclusions

EOC comprises biologically distinct histologic and molecular subtypes that differ in their mechanisms of treatment resistance and therapeutic vulnerabilities. In HGSOC, the strongest clinical evidence supports the molecularly guided use of PARP inhibitors and antiangiogenic therapy, whereas selected antibody–drug conjugates provide clinically validated options in target-positive disease. MAPK-directed therapy represents an important subtype-specific strategy in LGSOC. Treatment options for clear cell, endometrioid, and mucinous carcinomas remain less well defined and should reflect their distinct molecular profiles. HIPEC may provide benefit in appropriately selected clinical settings. By contrast, most metabolic, epigenetic, cellular, gene-editing, drug-delivery, and experimental physical approaches remain in early clinical stages or are predominantly preclinical. Further progress will require histotype- and context-specific patient selection, dynamic reassessment of resistance mechanisms and therapeutic targets, and rational treatment sequencing and combination strategies.

## Figures and Tables

**Table 1:** Molecular characteristics and heterogeneity of major ovarian carcinoma histologic subtypes.

Histologic Subtype	Frequency	Typical Mutations/Molecular Features	Degree of Heterogeneity	Refs.
HGSOC	~70%	*TP53* (96%), *BRCA1/2*, *HRD* (~50%), *CCNE1* and *MYC* amplification	Very high	[1,2,3]
Endometrioid	~10%	*CTNNB1*, *PIK3CA*, *ARID1A*, *PTEN*, *MSI*	Moderate	[4]
Clear cell	~10%	*ARID1A*, *PIK3CA*, *KRAS*, *PTEN* loss	Moderate	[4]
LGSOC	~5%	*KRAS*, *BRAF*, *NRAS*, *ERBB2*	Low-to-moderate	[4]
Mucinous	~3–4%	*KRAS*, *HER2*, molecular profile resembling colorectal cancer	Low	[4]

Abbreviations: HGSOC, high-grade serous carcinoma; LGSOC, low-grade serous carcinoma; HRD, homologous recombination deficiency; MSI, microsatellite instability.

**Table 2:** Integrated mapping of resistance mechanisms, therapeutic strategies, molecular context, clinical setting, and evidence maturity in EOC.

Resistance Mechanism or Therapeutic Vulnerability	Therapeutic Strategy	Histologic, Biomarker, and Clinical Context	Evidence Maturity and Major Limitations	Key References
VEGF-dependent angiogenesis and adaptive vascular escape	Bevacizumab; selected VEGFR tyrosine-kinase inhibitors; bevacizumab retreatment in a defined population	Advanced or recurrent EOC. No validated predictive biomarker. Retreatment evidence applies to platinum-sensitive recurrence after prior first-line bevacizumab.	Bevacizumab has randomized phase III support. Resistance may involve alternative proangiogenic signaling, hypoxia/HIF-1α, vessel co-option, and stromal remodeling. Retreatment should not be generalized beyond the studied population.	[27,28,29,30,31,32,33,34,35,36]
Homologous-recombination deficiency and DNA-repair dependence	PARP inhibitors (olaparib, niraparib, rucaparib); olaparib plus bevacizumab	Predominantly HGSOC; greatest benefit in BRCA1/2-mutated or HRD-positive tumors. Primarily first-line maintenance, with use determined by current indications and prior therapy.	Multiple randomized phase III trials and regulatory approvals. Resistance includes BRCA1/2 reversion, drug efflux, alternative repair, and replication-fork stabilization; some mechanisms may reduce subsequent platinum sensitivity.	[1,37,38,39,40,41,42,43,44]
FRα overexpression in platinum-resistant disease	Mirvetuximab soravtansine; mirvetuximab plus bevacizumab	FRα-expressing EOC, particularly HGSOC. MIRASOL required high FRα expression (≥75% of tumor cells with staining intensity ≥ 2+).	Randomized phase III evidence and regulatory approval for FRα-high platinum-resistant EOC. Limitations include ocular toxicity, antigen heterogeneity, target change over time, and acquired ADC resistance.	[45,46,47,48,49,50,51,52]
HER2 expression as an ADC target	Trastuzumab deruxtecan	Selected adults with unresectable or metastatic HER2-positive solid tumors, defined for the tumor-agnostic FDA indication as IHC 3+, after prior systemic treatment and without satisfactory alternative treatment options. This is not an EOC-specific approval.	Tumor-agnostic accelerated approval based on response rate and response duration rather than an EOC-specific randomized trial. EOC-specific efficacy, optimal testing strategy, and the effects of spatial and temporal HER2 heterogeneity remain insufficiently defined.	[1,49,52]
Replication stress and checkpoint dependence	WEE1 inhibition (adavosertib, ZN-c3) and ATR inhibition (berzosertib)	Investigated mainly in recurrent and platinum-resistant EOC. No validated routine biomarker is specified in the manuscript.	Phase I-II evidence. Adavosertib combinations showed activity, but development was limited by hematologic toxicity; berzosertib plus gemcitabine improved PFS in a randomized phase II trial, but the final analysis did not demonstrate a statistically significant OS benefit in the overall population. Exploratory subgroup findings require validation.	[53,54]
MAPK-pathway activation	Trametinib; avutometinib plus defactinib; binimetinib	Recurrent LGSOC. KRAS mutation is required for the FDA-approved avutometinib–defactinib combination, but not for trametinib. No validated selection biomarker has been established for binimetinib.	Randomized phase II/III evidence supports the use of trametinib in recurrent LGSOC. Avutometinib plus defactinib has accelerated FDA approval for previously treated KRAS-mutated recurrent LGSOC, pending confirmatory evidence. Binimetinib did not improve PFS in the phase III MILO/ENGOT-ov11 trial. Clinical activity should therefore not be interpreted as a uniform class effect.	[55,56,57,58]
Immune escape in biomarker-defined EOC	Pembrolizumab or dostarlimab; paclitaxel plus pembrolizumab with or without bevacizumab; ipilimumab plus nivolumab in the specified clinical settings	Recurrent or advanced MSI-H/dMMR or TMB-H EOC; PD-L1-positive platinum-resistant EOC for the paclitaxel–pembrolizumab regimen; platinum-resistant clear cell carcinoma for ipilimumab plus nivolumab.	Current recommendations support immunotherapy in selected biomarker- or histology-defined populations. The indications, strength of evidence, and regulatory status differ among regimens and should not be regarded as equivalent.	[53,59,60]
Immunosuppressive microenvironment and limited immune responsiveness in unselected EOC	PD-1/PD-L1 inhibition alone or combined with chemotherapy or bevacizumab	Predominantly unselected first-line, maintenance, recurrent, or platinum-resistant EOC populations; no validated universal predictive biomarker.	Checkpoint-inhibitor monotherapy has shown low response rates, and several phase III trials in predominantly unselected populations were negative. PD-L1 expression has not provided consistent patient selection across studies.	[61,62,63,64,65]
Surface-antigen expression with antigen heterogeneity and immune escape	CAR-T strategies targeting MSLN, MUC16, FRalpha, HER2, B7-H3, or dual targets	Antigen-positive EOC; predominantly preclinical and early clinical investigation. No validated clinical selection framework.	Mostly preclinical evidence with limited early clinical experience. Shared barriers include heterogeneous antigen expression, limited trafficking and persistence, immunosuppressive ascites/TME, extracellular-matrix barriers, CRS, and neurotoxicity.	[66,67,68,69,70,71,72,73,74,75,76,77]
Glycolytic/OXPHOS plasticity and compensatory metabolic switching	GLUT1, hexokinase, PFKFB3, and OXPHOS inhibitors; dual metabolic blockade including metformin-based combinations	HGSOC often shows OXPHOS dependence, whereas clear cell carcinoma shows enhanced glycolysis and glycogen accumulation. No validated clinical biomarker.	Predominantly preclinical evidence. Translation is limited by metabolic plasticity, intratumoral heterogeneity, compensatory nutrient use, and lack of validated patient-selection biomarkers.	[78,79,80,81,82,83,84]
Glutamine dependence and glutathione-mediated treatment resistance	ASCT2 or GLS1 inhibition, including BPTES, CB-839, Compound 968, and IACS-6274	Investigational contexts include MYC-driven tumors, ARID1A-deficient clear cell carcinoma, platinum-resistant EOC, KRAS-mutant tumors, and ASNS loss; none are a validated routine selection criterion.	Predominantly preclinical, with early phase I signals. Limitations include variable glutamine dependence, metabolic compensation, bioavailability, immune effects, and the need for predictive biomarkers.	[85,86,87,88,89,90,91,92,93,94,95]
Epigenetic silencing of tumor-suppressor and DNA-repair genes	DNMT inhibition with azacitidine, decitabine, or guadecitabine; combinations with platinum, PARP inhibition, or immunotherapy	Recurrent or platinum-resistant EOC; no validated routine predictive biomarker. Methylation and immune-response signatures remain exploratory.	Preclinical and phase I-II evidence with inconsistent clinical results. Limitations include schedule-dependent efficacy, hematologic toxicity, and lack of validated patient-selection markers.	[96,97,98,99,100,101,102]
Peritoneal dissemination in selected surgically resectable disease	Cytoreductive surgery plus HIPEC	Stage III EOC after neoadjuvant chemotherapy and interval debulking; selected stage IV disease in guidelines; first late recurrence in CHIPOR. Selection is clinical and surgical rather than molecular.	Randomized phase III evidence in defined settings and category 2B guideline recommendation for interval debulking. Benefit is not uniform across all recurrent-disease analyses; morbidity and resectability require careful selection.	[59,103,104,105,106,107,108]
Peritoneal dissemination requiring repeated locoregional drug delivery	Pressurized intraperitoneal aerosol chemotherapy (PIPAC)	Advanced, recurrent, or platinum-resistant EOC with peritoneal dissemination. No validated molecular biomarker; serial histologic response can be assessed using PRGS.	Early clinical evidence from phase I, multicenter, observational, real-world, and limited randomized studies. Regimens and populations remain heterogeneous, and adequately powered prospective trials are needed.	[109,110,111,112,113]

The table summarizes only relationships explicitly described in the manuscript. Biomarkers labeled as investigational are not established clinical selection criteria. Key references correspond to the current numbering in the uploaded revised manuscript. ADC, antibody–drug conjugate; CAR-T, chimeric antigen receptor T cell; CRS, cytokine release syndrome; dMMR, mismatch repair deficient; EOC, epithelial ovarian cancer; FRα, folate receptor alpha; HIPEC, hyperthermic intraperitoneal chemotherapy; HGSOC, high-grade serous ovarian carcinoma; HRD, homologous recombination deficiency; LGSOC, low-grade serous ovarian carcinoma; MSI-H, microsatellite instability-high; OXPHOS, oxidative phosphorylation; PARP, poly(ADP-ribose) polymerase; PIPAC, pressurized intraperitoneal aerosol chemotherapy; PRGS, Peritoneal Regression Grading Score; TMB-H, tumor mutational burden-high; TME, tumor microenvironment.

## Data Availability

No new data were created or analyzed in this study. Data sharing is not applicable to this article.

## Abbreviations

The following abbreviations are used in this manuscript:

2-DG	2-deoxyglucose
3-BP	3-bromopyruvate
AAV	adeno-associated virus
ACC	acetyl-CoA carboxylase
ACC2	acetyl-CoA carboxylase 2
ACLY	ATP-citrate lyase
ADC	antibody–drug conjugate
ADI-PEG20	pegylated arginine deiminase
AGP	α1-acid glycoprotein
aHR	adjusted hazard ratio
AKT	protein kinase B
AML	acute myeloid leukemia
AMP	adenosine monophosphate
AMPK	AMP-activated protein kinase
AOA	aminooxyacetate
ASCT2	alanine-serine-cysteine transporter 2
ATP	adenosine triphosphate
ATR	ataxia telangiectasia and Rad3-related protein
AZA	azacitidine
BER	base excision repair
BET	bromodomain and extraterminal
BPTES	bis-2-(5-phenylacetamido-1,3,4-thiadiazol-2-yl)ethyl sulfide
CA-125	cancer antigen 125
CAF	cancer-associated fibroblast
CAR	chimeric antigen receptor
CAR-NK	chimeric antigen receptor natural killer cell
CAR-T	chimeric antigen receptor T cell
CCC	clear cell carcinoma
CI	confidence interval
CIN	chromosomal instability
CPS	combined positive score
CRISPR/Cas9	clustered regularly interspaced short palindromic repeats/CRISPR-associated protein 9
CRS	cytokine release syndrome
CSC	cancer stem cell
DAC	decitabine
DAMP	danger-associated molecular pattern
DC	dendritic cell
DCA	dichloroacetate
DCR	disease control rate
DNMT	DNA methyltransferase
DNMTi	DNA methyltransferase inhibitor
DON	6-diazo-5-oxo-L-norleucine
DSB	double-strand break
dsRNA	double-stranded RNA
EC	endometrioid carcinoma
ECM	extracellular matrix
EMT	epithelial–mesenchymal transition
EOC	epithelial ovarian cancer
EPR	enhanced permeability and retention
ERK	extracellular signal-regulated kinase
ERV	endogenous retrovirus
EV	extracellular vesicle
EZH2	enhancer of zeste homolog 2
FAO	fatty acid β-oxidation
FASN	fatty acid synthase
FDA	U.S. Food and Drug Administration
FDG-PET/CT	fluorodeoxyglucose positron emission tomography/computed tomography
FGF	fibroblast growth factor
FIGO	International Federation of Gynecology and Obstetrics
FRα	folate receptor alpha
GLS1	glutaminase 1
GLUT1	glucose transporter 1
GM-CSF	granulocyte-macrophage colony-stimulating factor
GOG	Gynecologic Oncology Group
GPX4	glutathione peroxidase 4
GR	glucocorticoid receptor
GSH	glutathione
H-FIRE	high-frequency irreversible electroporation
HA	hyaluronic acid
HDAC	histone deacetylase
HDACi	histone deacetylase inhibitor
HER2	human epidermal growth factor receptor 2
HGF	hepatocyte growth factor
HGSOC	high-grade serous ovarian carcinoma
HIF-1α	hypoxia-inducible factor 1 alpha
HIPEC	hyperthermic intraperitoneal chemotherapy
HR	hazard ratio
HRD	homologous recombination deficiency
HRP	homologous recombination-proficient
HRR	homologous recombination repair
HSP70	heat shock protein 70
HSV	herpes simplex virus
ICD	immunogenic cell death
ICG	indocyanine green
ICI	immune checkpoint inhibitor
IDS	interval debulking surgery
IFN	interferon
IL	interleukin
IRE	irreversible electroporation
LDHA	lactate dehydrogenase A
LGSOC	low-grade serous ovarian carcinoma
LNP	lipid nanoparticle
LPA	lysophosphatidic acid
LTR	long terminal repeat
MAPK	mitogen-activated protein kinase
MC	mucinous carcinoma
MDS	myelodysplastic syndrome
MDSC	myeloid-derived suppressor cell
mEHT	modulated electrohyperthermia
MEK	mitogen-activated protein kinase kinase
MET	mesenchymal–epithelial transition
MHC	major histocompatibility complex
miR-497	microRNA-497
MIRV	mirvetuximab soravtansine
MMT	mesothelial–mesenchymal transition
MOSE	mouse ovarian surface epithelial
MSI	microsatellite instability
MSI-H/dMM	microsatellite instability-high/mismatch repair-deficient
MSLN	mesothelin
mTOR	mechanistic target of rapamycin
MWA	microwave ablation
NAD+	nicotinamide adenine dinucleotide
NADH	reduced nicotinamide adenine dinucleotide
NADPH	reduced nicotinamide adenine dinucleotide phosphate
NAMPT	nicotinamide phosphoribosyltransferase
NCCN	National Comprehensive Cancer Network
NF-κB	nuclear factor kappa B
NK	natural killer
NKT	natural killer T cell
OCLM	ovarian cancer liver metastasis
OCSC	ovarian cancer stem cell
ORR	objective response rate
OS	overall survival
OXPHOS	oxidative phosphorylation
PARP	poly(ADP-ribose) polymerase
PARPi	poly(ADP-ribose) polymerase inhibitor
PCI	Peritoneal Cancer Index
PD-1	programmed cell death protein 1
PD-L1	programmed death-ligand 1
PDGF	platelet-derived growth factor
PDT	photodynamic therapy
PDX	patient-derived xenograft
PEG	polyethylene glycol
PEI	polyethyleneimine
PFH	perfluorohexane
PFKFB3	6-phosphofructo-2-kinase/fructose-2,6-bisphosphatase 3
PFS	progression-free survival
PHGDH	phosphoglycerate dehydrogenase
PI3K	phosphoinositide 3-kinase
PIPAC	pressurized intraperitoneal aerosol chemotherapy
PKM2	pyruvate kinase M2
PLD	pegylated liposomal doxorubicin
PLGA	poly(lactic-co-glycolic acid)
PMC	peritoneal mesothelial cell
PRC2	polycomb repressive complex 2
PRGS	Peritoneal Regression Grading Score
PSDT	photo-sonodynamic therapy
R0	no macroscopic residual disease
RCT	randomized controlled trial
RE	repeat element
RFA	radiofrequency ablation
RIG-I	retinoic acid-inducible gene I
RNA	ribonucleic acid
RNP	ribonucleoprotein
ROS	reactive oxygen species
SAHA	suberoylanilide hydroxamic acid
SASP	senescence-associated secretory phenotype
SCD1	stearoyl-CoA desaturase 1
scFv	single-chain variable fragment
SDT	sonodynamic therapy
SHMT	serine hydroxymethyltransferase
SLN	solid lipid nanoparticle
SOAT1	sterol O-acyltransferase 1
SSB	single-strand break
STIC	serous tubal intraepithelial carcinoma
SWI/SNF	switch/sucrose non-fermentable
T-DXd	trastuzumab deruxtecan
TAM	tumor-associated macrophage
TCA	tricarboxylic acid
TGF-β	transforming growth factor beta
TIC	tumor-initiating cell
TIS	therapy-induced senescence
TLR3	Toll-like receptor 3
TMB-H	tumor mutational burden-high
TME	tumor microenvironment
TNF	tumor necrosis factor
TP	triptolide
Treg	regulatory T cell
TRX	thioredoxin
uPAR	urokinase-type plasminogen activator receptor
VEGF	vascular endothelial growth factor
VEGFR	vascular endothelial growth factor receptor
VISTA	V-domain immunoglobulin suppressor of T-cell activation
VPA	valproic acid
WEE1	WEE1 G2 checkpoint kinase
xCT	cystine/glutamate antiporter
